# Glioblastoma stem cells deliver ABCB4 transcribed by ATF3 via exosomes conferring glioblastoma resistance to temozolomide

**DOI:** 10.1038/s41419-024-06695-6

**Published:** 2024-05-06

**Authors:** Xiangdong Xu, Yaofeng Zheng, Linting Luo, Zhongsheng You, Huajian Chen, Jihui Wang, Fabing Zhang, Yang Liu, Yiquan Ke

**Affiliations:** 1 Department of Neuro-oncological Surgery, Zhujiang Hospital, Southern Medical University, Guangzhou, 510282 PR China; 2 The Neurosurgery Institute of Guangdong Province, Zhujiang Hospital, Southern Medical University, Guangzhou, 510282 PR China; 3 Department of Neurology, Liwan Central Hospital of Guangzhou, Guangzhou, PR China

**Keywords:** CNS cancer, Cancer stem cells

## Abstract

Glioblastoma stem cells (GSCs) play a key role in glioblastoma (GBM) resistance to temozolomide (TMZ) chemotherapy. With the increase in research on the tumour microenvironment, exosomes secreted by GSCs have become a new focus in GBM research. However, the molecular mechanism by which GSCs affect drug resistance in GBM cells via exosomes remains unclear. Using bioinformatics analysis, we identified the specific expression of ABCB4 in GSCs. Subsequently, we established GSC cell lines and used ultracentrifugation to extract secreted exosomes. We conducted in vitro and in vivo investigations to validate the promoting effect of ABCB4 and ABCB4-containing exosomes on TMZ resistance. Finally, to identify the transcription factors regulating the transcription of ABCB4, we performed luciferase assays and chromatin immunoprecipitation-quantitative PCR. Our results indicated that ABCB4 is highly expressed in GSCs. Moreover, high expression of ABCB4 promoted the resistance of GSCs to TMZ. Our study found that GSCs can also transmit their highly expressed ABCB4 to differentiated glioma cells (DGCs) through exosomes, leading to high expression of ABCB4 in these cells and promoting their resistance to TMZ. Mechanistic studies have shown that the overexpression of ABCB4 in GSCs is mediated by the transcription factor ATF3. In conclusion, our results indicate that GSCs can confer resistance to TMZ in GBM by transmitting ABCB4, which is transcribed by ATF3, through exosomes. This mechanism may lead to drug resistance and recurrence of GBM. These findings contribute to a deeper understanding of the mechanisms underlying drug resistance in GBM and provide novel insights into its treatment.

## Introduction

Glioblastoma (GBM) is the most common primary malignant central nervous system (CNS) tumour [1–3], accounting for 14.6% of all CNS tumours and 48.3% of all malignant CNS tumours [4] with an average incidence rate of 3.22/100,000 people [5]. However, GBM generally has a poor prognosis and short survival time, with a median survival time of only 14.6–16.6 months [5, 6]. Despite the development of multimodal treatment strategies, including surgical resection, chemotherapy, and radiation therapy, the outcomes of patients with GBM remain unsatisfactory [7, 8]. Temozolomide (TMZ) is the most effective chemotherapeutic drug for the treatment of GBM; however, its effectiveness is limited by drug resistance [9]. Therefore, there is an urgent need to determine the underlying TMZ resistance mechanisms to improve TMZ efficacy [10].

GBM stem cells (GSCs) are a minimised glioma cell subpopulation characterised by their stem cell properties [11, 12]. GSCs represent a distinct tumour cell population that can proliferate, self-renew, differentiate, initiate, and maintain tumours [13]. Therefore, GSCs are the main source of cells that initiate and maintain glioma growth and play a decisive role in GBM recurrence [14, 15]. Furthermore, GSCs play an important role in TMZ chemotherapy resistance [16]; however, the specific mechanism requires further study.

GBM cells participate in TMZ therapy resistance through multidrug resistance mechanisms, in which the ATP-binding cassette (ABC) transporter superfamily plays a key role [17, 18]. Several ABC transporter superfamily members were found to be significantly overexpressed in CD133 positive GSCs [19, 20]. In addition, overexpression of ABCB1 and ABCG2 in GSCs has been associated with GSC resistance to chemotherapeutic agents [21]. These studies suggest that the ABC transporter superfamily may be involved in the drug resistance of GSCs.

ABCB4, a member of the ABC transporter superfamily that is highly similar in structure to ABCB1, is mainly expressed in the liver and mediates phosphatidylcholine transport [22]. Fischer et al. found that ABCB4 participates in cell resistance to chemicals [23]. A previous study revealed that ABCB4 is overexpressed in doxorubicin-resistant breast cancer cells, reduces chemotherapy drug sensitivity, and may cause drug resistance [24]. However, the biological role of ABCB4 in glioma has seldom been reported.

Exosomes, which are secreted by a variety of cells, are extracellular vesicles 40–150 nm in size with lipid bilayer membranes [25]. Increasing evidence shows that exosomes play an important role in tumour drug resistance by transferring ABC transporters between cells [26–28]. Concurrently, sequencing results suggest that ABCB4 is present in exosomes and plays a key role in chemical resistance [29]; however, the mechanism is still unclear and requires further study.

In this study, we examined ABCB4 expression in GSCs and found that ABCB4 is involved in TMZ resistance. We also found that GSCs can transfer ABCB4 to differentiated glioma cells (DGCs) through exosomes, thus promoting DGC resistance to TMZ. Additionally, through transcription factor analysis and luciferase assays, we found that ABCB4 overexpression in GSCs may be regulated by the transcription factor ATF3. Overall, our study reveals a novel mechanism for GSC resistance, suggesting that ABCB4 may be a potential therapeutic target in patients with GBM.

## Materials and methods

### Data collection

The datasets GSE98126 and GSE157506 were obtained from the Gene Expression Omnibus (GEO) database (https://www.ncbi.nlm.nih.gov/geo/). Two transcriptomic datasets of glioma, along with corresponding clinical data, designated ‘CGGA_mRNAseq_325’ and ‘CGGA_mRNAseq_693’, were acquired from the Chinese Glioma Genome Atlas (CGGA) database (available at http://www.cgga.org.cn/index.jsp). Similarly, the ‘TCGA_mRNAseq_698’ dataset, encompassing both TCGA-LGG and TCGA-GBM glioma transcriptomic data and corresponding clinical information, was sourced from The Cancer Genome Atlas (TCGA) database (https://portal.gdc.cancer.gov/). All datasets mentioned are derived from public databases. Therefore, the information extracted is exempt from further ethical approval.

### Data analysis

Patients without survival information or those with a survival period of <30 days were excluded from further analysis. Differential gene expression analysis was conducted using the DESeq2 package on transcriptome sequencing read count data. Differentially expressed genes (DEGs) were identified based on an adjusted *P* value threshold of <0.05 (Pval.adj < 0.05) and an absolute log2-fold change >1 (|log2(FC)| > 1). Univariate Cox proportional hazards regression analysis was performed with the survival package to identify the prognosis-related DEGs. These DEGs were further refined through least absolute shrinkage and selection operator (LASSO) regression in the glmnet package. Optimal penalty parameter λ selection was achieved through tenfold cross-validation, optimising variable selection. These variables were subsequently employed to construct a multivariate Cox proportional hazards model. This model enabled the calculation of patient risk scores based on gene expression values (Xi) and their corresponding coefficients (βi), using the following formula: riskscore = Σ(βi * Xi). Total cohorts were then divided into low- and high-risk groups using the cohort’s median risk score. The Kaplan–Meier (KM) method was utilised for the survival analysis, and survival R package performed the log-rank test. Heatmap visualisation was achieved using the heatmap package.

### GBM specimen and cell culture

Brain tumour samples were obtained from consenting patients diagnosed with gliomas. From 2016 to 2021, the Department of Neurosurgery at Zhujiang Hospital collected 40 glioma samples preserved in paraffin, along with their corresponding clinicopathological information, from patients who underwent surgical procedures.

For GBM stem cell culture, tumour samples were collected from consenting patients diagnosed as GBM. The obtained GBM tissue was mechanically segmented and digested by enzyme until it reached the single-cell state. Digested cells were cultured in Dulbecco’s modified Eagle medium (DMEM)/F12 (Gibco, USA) supplemented with epidermal growth factor (20 ng/ml, Peprotech, USA), basic fibroblast growth factor (20 ng/ml, Peprotech, USA), and B27 (1:50, Gibco, USA) for 2 weeks. The resultant spheroids were defined as glioma stem-like cells. Fresh neural stem cell medium was added to the GSCs every 2 days. Immunofluorescence assays were performed to identify GSCs markers, including CD133 and Nestin. GSCs expansion was performed using both adherent and suspension culture methods. GSCs were placed in a laminin-coated flask (10 μg/ml, Gibco, USA) in an adherent culture system and grown in serum-free medium. In the suspension culture system, stem cell spheres were generated and propagated, as previously described [30]. GSCs from passages 2–10 were used. To obtain DGCs, GSCs were cultured in DMEM supplemented with 10% foetal bovine serum (Gibco, USA). The cells were incubated at 37 °C with 5% CO_2_ and refreshed every 3 months using frozen reserves. The normal human astrocytes HA and HEB were purchased from the Chinese Academy of Sciences Cell Bank (Shanghai, China). Cell lines were cultured in high glucose DMEM medium (Gibco, USA) containing 10% foetal bovine serum (FBS, Gibco, USA).

### Immunohistochemistry and immunofluorescence staining

Tumour tissue samples from patients with glioma and xenograft samples of glioma were preserved using 4% formalin, embedded in paraffin, and then cut into 4 μm sections. After deparaffinization and dehydration, tissue sections were incubated in 3% hydrogen peroxide for 10 min, blocked with 5% bovine serum albumin (BSA) in phosphate buffered saline (PBS) for 1 h at room temperature, and the slides treated with primary antibodies against ABCB4 (Thermo Fisher Scientific, USA, PA5-78692), Ki67 (Abcam, UK, ab15580), and cleaved-caspase-3 (GeneTex, USA, GTX86952) at 4 °C overnight. Secondary antibodies were added and incubated for 1 h at room temperature. DAB staining was used to detect specific molecules, and the slides were counterstained with hematoxylin.

Immunofluorescence staining was performed using primary antibodies against CD133 (CST, USA, #64326), Nestin (CST, USA, #33475), and ABCB4. The immunostaining was carried out overnight at 4 °C, followed by incubation with fluorochrome-conjugated antibodies. Finally, DAPI was used to stain the cell nuclei. A fluorescence microscope or laser scanning confocal microscope (Nikon, Japan) was used to capture images.

### Sphere formation assay

Individually separated GSCs were placed in 24-well dishes and incubated in serum-free medium at 37 °C for 7 days. A fluorescence microscope (Leica, Germany) was used to capture images of five randomly chosen regions from each group.

### Extraction of RNA and quantitative reverse transcription polymerase chain reaction (qRT-PCR)

Total RNA was extracted from glioma cells using TRIzol reagent (Invitrogen, USA). An miRNeasy Mini Kit (Qiagen, Germany) was used to extract RNA from the exosomes. The PrimeScriptTMRT reagent kit (TaKaRa, Japan) was used to convert 1 μg total RNA into cDNA, with the assistance of the gDNA Eraserkit. For realtime-PCR, a SYBR®Premix Ex Taq^TM^ Kit (Tli RNaseH Plus, TaKaRa, Japan) was used. A LightCycler 480 quantitative PCR instrument (Roche, USA) was used. Relative RNA expression was determined using the ΔΔCt method. Sangon Biotech Ltd. (Shanghai, China) provided the primers (Supplementary Table S1).

### Western blot (WB) analysis

A Whole Cell Lysis Assay (KeyGEN BioTECH, China) was used to extract both total and exosome proteins. Protein extracts were isolated using 8–12% sodium dodecyl-sulfate polyacrylamide gel electrophoresis and subsequently transferred onto polyvinylidene fluoride membranes (Millipore, USA). Following the addition of 5% BSA (Sigma-Aldrich, USA), the membranes were incubated with primary antibodies (Supplementary Table S2) for 12 h at 4 °C. The membranes were then incubated with secondary antibodies conjugated to horseradish peroxidase for 1 h at ambient temperature. Enhanced chemiluminescence (ECL, Millipore, USA) was used to visualise the protein bands. ImageJ software was used to analyse the strength of the protein bands, which were normalised to those of GAPDH. The full, uncropped western blots can be found in the Supplementary Material (Fig. S7).

### Infection with lentiviruses and transfection with siRNA

A lentivirus containing ABCB4 short hairpin RNA was constructed by Hanbio (Shanghai, China). The cells were cultured with the lentivirus and polybrene for 24 h. Stable control and specific knockdown cells were selected and maintained in the presence of puromycin (2 μg/mL, Solarbio, China). Hanbio provided the siRNA for ATF3 and negative control sequences, which were then transfected using the lipofectamine® 3000 reagent (Cat# L3000015, Thermo Fisher Scientific, USA).

### Drugs

TMZ powder (Sigma-Aldrich, USA) was dissolved in dimethyl sulfoxide (Sigma-Aldrich, USA) to a final concentration of 100 mM.

### Assessment of cellular viability

Cell viability was determined using a CCK-8 assay. Cells were seeded in a 96-well dish at 3 × 10^3^ cells/well and incubated for 24 h. Following exposure to various concentrations of TMZ for 48 h, or incubation in 200 μM TMZ for several days, CCK-8 solution (Dojindo, Japan) was added. Cell viability was measured at 450 nm using a microplate reader (BioTek, USA).

Cell viability was analysed using Colony forming assay (CFA). Exponentially growing cells were seeded into a 6-well plate (1000 cells/well) with or without the addition of TMZ. After incubation for 10–15 days, colonies in the wells were stained with 0.5% crystal violet for 30 min. Subsequently, the number of colonies was observed and counted using a microscope.

### Flow cytometry

Apoptosis was detected using the Annexin V-FITC kit (KeyGEN BioTECH, China). Cells were exposed to TMZ (200 μM) for 48 h and subsequently suspended in a binding buffer that included annexin V and propidium iodide. Following a 15-min incubation in the absence of light, the apoptosis rate was assessed using a FACSCalibur device (BD Bioscience, USA).

### In vivo xenograft assay

Male nude BALB/c mice, aged 5–8 weeks, were acquired from the Central Animal Facility at Southern Medical University. The study protocols were approved by the Animal Care and Use Committee of the Southern Medical University. BALB/c nude mice were stereotactically injected with GSC-Luc stably transfected with Lentivirus-shABCB4 (ABCB4 KD) or lentivirus-vector control (Ctrl KD) into the right hemicerebrum. Each treatment group consisted of 15 mice. Starting on the 7th day post-transplantation, mice carrying the tumour were administered TMZ at 20 mg/kg/day, 5 days/week, for three cycles. Tumour growth was observed using an in vivo imaging device (IVIS Lumina II, USA) following administration of the luciferase substrate d-luciferin (Yeasen, China) via intraperitoneal injection. After 4 weeks, seven nude mice were euthanized in each group. Brains were collected and immunohistochemically stained. The survival curves were plotted for the remaining mice. In addition, to verify the stability and reliability of the experimental results, we used another 30 mice and repeated the above experimental procedure, but we did not give TMZ treatment this time.

### Co-culture assay

GSCs and DGCs were co-cultured in a Transwell plate (Corning, USA) with a 0.4-μm polycarbonate filter for 48 h at a 1:1 ratio. The upper chamber contained GSCs, whereas the lower chamber contained DGCs.

### Exosome extraction and purification

Since the culture medium for GSCs does not require the addition of serum, the cells are directly removed through centrifugation, and the cell culture medium is subsequently retained. In contrast, DGCs require serum-based culture medium. Therefore, the exosomes were removed from FBS by ultracentrifugation at 100,000 × *g* for 8 h (Exo-free-FBS). When the confluence of DGCs reached ~80%, DMEM containing 10% Exo-free-FBS was added, and the cells were incubated for 48 h at 37 °C and 5% CO_2_ before collecting the cell culture medium.

The collected cell culture medium was then centrifuged at 300 × *g* for 10 min at 4 °C. The supernatant was collected and centrifuged again at 2000 × *g* for 15 min at 4 °C, followed by another centrifugation at 5000 × *g* for 15 min. The supernatant was then collected and centrifuged at 12,000 × *g* for 30 min. The final supernatant was collected and ultracentrifuged at 100,000 × *g* for 70 min at 4 °C (Beckman Coulter, Brea, USA). The exosome pellets were washed with sterilised PBS and ultracentrifuged at 100,000 × *g* for 1 h at 4 °C. Subsequently, exosomes were resuspended in 100 μL of PBS and stored at −80 °C until use.

### Exosome characterisation

Exosome morphology was analysed using transmission electron microscopy (TEM, Hitachi HT7650, Japan). To determine the size distribution of the isolated exosomes, a Zetasizer Nano-Zs (Malvern Panalytical, UK) was used in accordance with the manufacturer’s guidelines. A bicinchoninic acid (BCA) protein assay kit (KeyGEN BioTECH, China) was used to determine the concentration of the isolated exosomes according to the manufacturer’s instructions. To examine the protein indicators of the exosomes, WB was performed using antibodies against CD9, ALIX, CD63, and calnexin.

### Exosome uptake assay

GSC exosomes were labelled with fluorescent PKH26 using a cell linker kit (Sigma-Aldrich, USA) to observe exosome trafficking. The exosomes labelled with PKH26 were rinsed with PBS, centrifuged at 100,000 × *g* for 20 min at 4 °C, and the exosomes suspended in PBS. After labelling with PKH26, the exosomes (10 μg/ml) were co-cultured with DGCs for 24 h, and a confocal fluorescence microscope used to observe the absorption of exosomes by DGCs.

### Luciferase reporter assay

Before transfection, 1 × 10^5^ GSCs were seeded in a 24-well dish. The following day, cells were co-transfected with the control pcDNA3.1 empty vector (pcDNA3.1, 225 ng) or ATF3 plasmid (225 ng)-based firefly luciferase reporter and the *Renilla* luciferase control vector pGL4.73 (50 ng, Promega, USA) using the K-2 transfection reagent (Biontex, Germany). Following 48-h incubation, the *Renilla* levels and firefly luciferase activities were assessed in accordance with the manufacturer’s guidelines (Promega, USA). Relative values were obtained by normalising firefly luciferase values to *Renilla* luciferase values.

### Chromatin immunoprecipitation (ChIP)

Following the cross-linking and lysis of cells, DNA fragments were sonicated on ice to achieve lengths ranging from 200 to 1000 base pairs. Protein–DNA complexes were precipitated using an Anti-ATF3 antibody (CST, USA, #33593) and control IgG. Procedures were performed in accordance with the Pierce Agarose ChIP Kit guidelines (Thermo Scientific, USA). Immunoprecipitated DNA and the corresponding input DNA were subjected to reverse cross-linking at 65 °C overnight. This DNA was then used in a qPCR assay with the SYBR Green qPCR mix to investigate the potential ATF3-binding locations in the ABCB4 promoter, employing specific primers (Fig. S6).

### Statistical analyses

GraphPad Prism 6 (GraphPad Software Inc., USA) was used for all statistical analyses. The data is displayed as the average ± standard error from three separate trials. Statistical significance was evaluated using either Student’s *t*-test or one-way analysis of variance with Bonferroni correction to account for multiple comparisons. Survival rates were calculated utilising the Kaplan–Meier technique, and variations in mortality were assessed through the log-rank test. Significance was determined for all tests using a two-sided approach, with *P* values < 0.05 considered significant.

## Results

### ABCB4 expression was upregulated in TMZ-treated GSCs, and ABCB4 was strongly associated with the prognosis of GBM

**Fig. 1:**
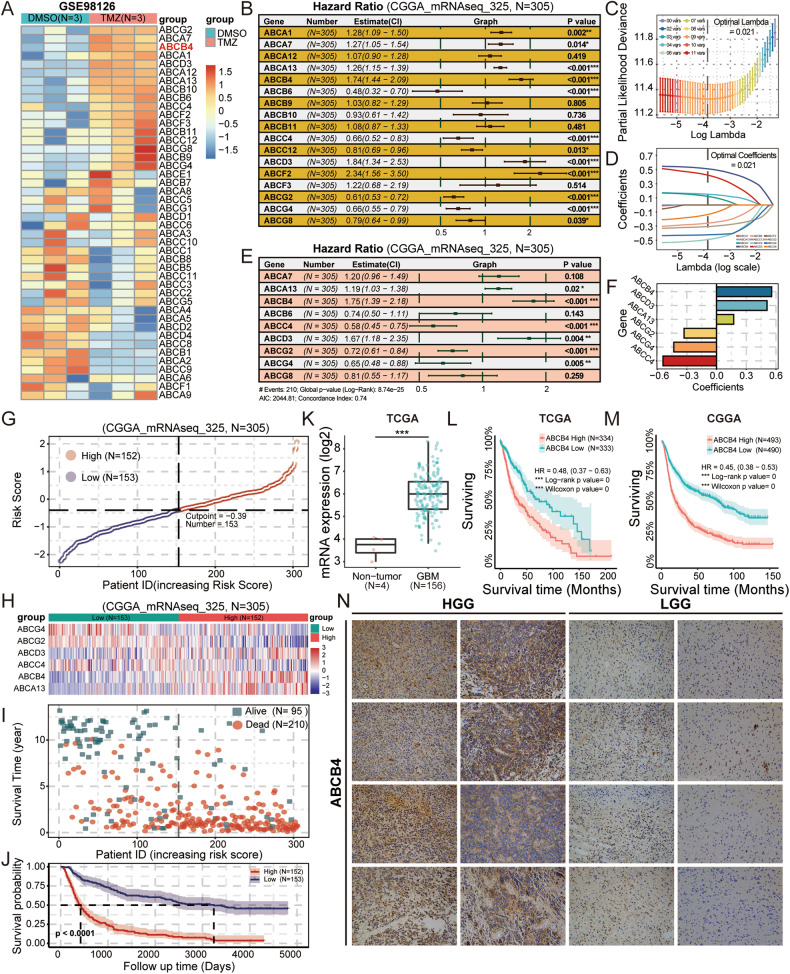
ABCB4 is highly expressed in GBM and is closely related to poor prognosis. **A** Heatmap showing the expression profile of the ABC transporter family in the GSE98126 dataset (Group DMSO = 3, Group TMZ = 3). **B** Forest plot of univariate Cox proportional hazards regression analysis for the upregulated members of the ABC transporter family (CGGA_mRNAseq_325, N = 305). **C** Tenfold cross-validation used to determine the optimal λ value. **D** Coefficient profiles of the LASSO model. **E** Forest plot of multivariate Cox proportional hazards regression analysis of the best members from the LASSO model (CGGA_mRNAseq_325, N = 305). **F** Variables identified by the multivariate Cox model and their corresponding coefficients. **G** Stratification into high-risk (N = 153) and low-risk (N = 152) groups based on the median overall risk score. **H** Expression profiles of individual variables in the model across high-risk (N = 153) and low-risk (N = 152) groups. **I** Distribution plot of survival status (Group Alive = 95, Group Dead = 210). **J** Kaplan–Meier survival curves between the high-risk (N = 153) and low-risk (N = 152) groups (CGGA_mRNAseq_325, N = 305). **K** The box plot in TCGA database shows the expression of ABCB4 between normal tissue (red) and GBM (green). **L** Prognosis efficiency of ABCB4 in WHO grade patients within TCGA RNA-seq data. **M** Prognosis efficiency of ABCB4 in WHO grade patients within the CGGA database. **N** Immunohistochemical staining of ABCB4 in HGG and LGG samples. Scale bar = 100 μm. *p < 0.05; **p < 0.01; ***p < 0.001; ns not significant.

To explore the key role of the ABC transporter family in GSCs resistance to TMZ, we screened GSE98126 datasets from the GEO database. This data set records in detail the cellular response and transcriptome changes of GSCs to TMZ treatment. Through the detailed analysis of differential gene expression in the GSE98126 data set, we found that 17 members of the ABC transporter family showed a trend of upregulation in TMZ-treated GSCs (Fig. 1A). This upregulation may be associated with drug resistance in GSCs. To elucidate the clinical implications of these upregulated genes, we used the mRNAseq_325 dataset from the CGGA database [31]. Univariate Cox proportional hazards regression analysis identified 12 genes that correlated with patient prognosis (Fig. 1B). To establish a precise prediction model, we used tenfold cross-validation LASSO regression analysis (Fig. 1C, D) and multivariate Cox proportional hazard regression analysis (Fig. 1E). Ultimately, we identified an optimal model comprising of six molecules (Fig. 1F). Subsequently, using the model equation, we calculated patient risk scores and categorised patients into high- and low-risk groups based on the median values of these risk scores (Fig. 1G–I). Survival analysis revealed that patients in the high-risk group had poorer prognosis (Fig. 1J). To corroborate the reliability of our analysis, we performed a similar analysis on another transcriptomic dataset, mRNA_693, from the CGGA (Fig. S1A–I) and the glioma dataset from TCGA (Fig. S2A–I). Consistent outcomes across these datasets further substantiated the credibility of our findings. Notably, ABCB4 exhibited consistent behaviour across all three datasets, suggesting a potentially widespread and significant biological role in glioma resistance. Furthermore, to firmly establish the pivotal role of ABCB4 in GBM, we replicated the aforementioned analysis using 84 GBM samples from the CGGA’s mRNAseq_325 dataset (Fig. S3A–I). Delightfully, the results of this replication analysis echoed our previous findings, reinforcing the strong association between ABCB4 and the prognosis of GBM patients. Therefore, we focused on ABCB4 in this study. To further investigate the clinical significance of ABCB4 in GBM, we analysed its expression in the TCGA and CGGA databases. First, data from TCGA showed that ABCB4 was highly expressed in GBM tissues compared to normal tissues (Fig. 1K). Further, the TCGA (Fig. 1L) and CGGA (Fig. 1M) data suggested that high ABCB4 expression was associated with poor prognosis. To validate the results of the bioinformatics analysis, high-grade glioma (HGG) and low-grade glioma (LGG) samples were subjected to an immunohistochemistry assay. The results showed that ABCB4 expression was upregulated in HGG tissues compared to LGG tissues (Fig. 1N). Overall, these findings indicate that ABCB4 is upregulated in GBM and closely related to the prognosis of patients with GBM. Moreover, ABCB4 may be associated with drug resistance in GSCs.

### ABCB4 was highly expressed in GSCs

**Fig. 2:**
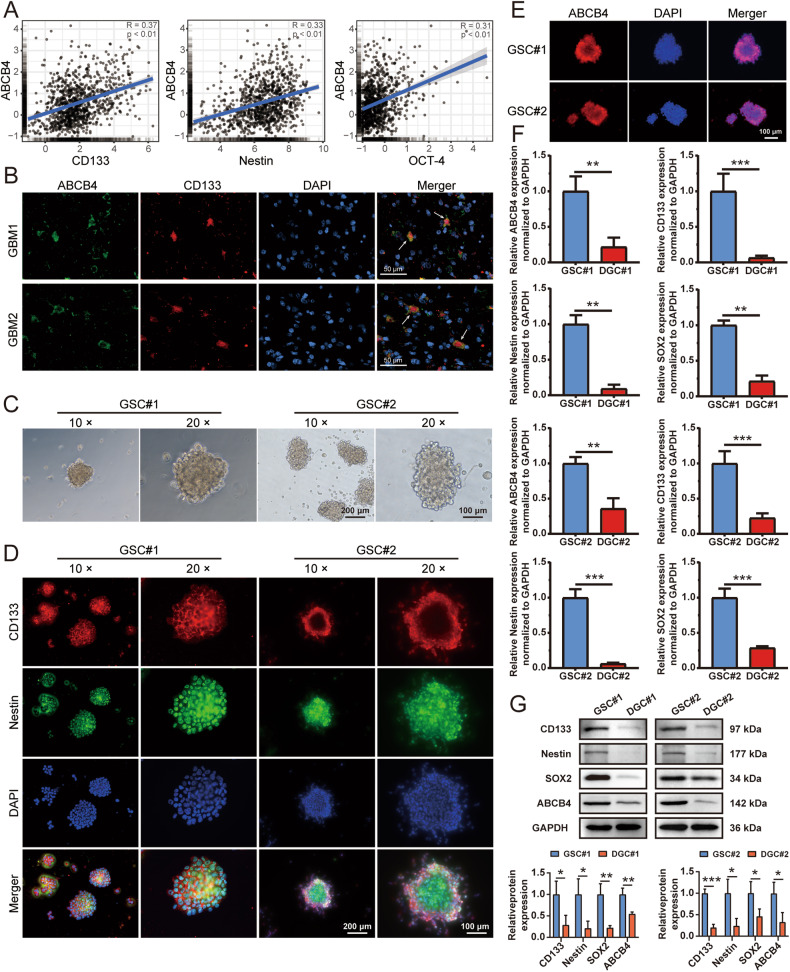
ABCB4 is highly expressed in GSCs. **A** Correlation between gene expression of ABCB4 and stem cell markers in CGGA glioma dataset. **B** Localisation of ABCB4 (green) and CD133 (red) examined by immunofluorescence confocal microscopy. Nuclei stained with DAPI (blue). Scale bar = 50 μm. **C** Representative images of GSC#1 and GSC#2 cultured in serum-free medium to form floating spheres. Scale bar = 100/200 μm. **D** Fluorescence staining for CD133 (red) and Nestin (green) in GSCs. Nuclei stained with DAPI (blue). Scale bar = 100/200 μm. **E** Fluorescence staining for ABCB4 (red) in GSCs. Nuclei stained with DAPI (blue). Scale bar = 100 μm. **F** Detection of ABCB4, CD133, Nestin, and SOX2 mRNA levels in GSCs and DGCs via qRT-PCR. **G** Detection of ABCB4, CD133, Nestin, and SOX2 protein levels in GSCs and DGCs via WB. All experiments were repeated independently three times. Data are presented as mean ± standard deviation. *p < 0.05; **p < 0.01; ***p < 0.001; ns not significant.

To further investigate the relationship between ABCB4 and GSCs, we analysed the correlation between ABCB4 and the GSC markers CD133, Nestin, and OCT-4 using the CGGA [32]. These findings indicated a positive correlation between ABCB4 and CD133, Nestin, and OCT-4, suggesting a potential association between ABCB4 expression and GSCs (Fig. 2A). Subsequently, the co-localisation of ABCB4 and CD133, a recognised marker of GSCs, was examined in glioblastoma tissues using confocal microscopy. The results demonstrated the co-localisation of ABCB4 and CD133 (Fig. 2B), thus supporting the notion that GSCs express ABCB4. Human GSCs isolated and cultured from glioblastoma patient specimens were designated GSC#1 and GSC#2. Both GSC#1 and GSC#2 formed tumour spheres in serum-free medium (Fig. 2C). Immunofluorescence staining indicated that the GSC markers CD133 and Nestin were expressed in GSC#1 and GSC#2 neurospheres (Fig. 2D). These results suggested that we successfully obtained two strains of GSCs. Immunofluorescence assay confirmed that ABCB4 was expressed in GSCs (Fig. 2E). GSCs differentiate in response to serum treatment. The differentiation status of cells was determined by assessing the expression of glial fibrillary acidic protein (GFAP). GFAP expression is minimal in undifferentiated GSCs, but significantly increases upon differentiation (Fig. S4A). Next, we investigated ABCB4 expression in DGCs and found that ABCB4 was significantly lower in DGCs than in GSCs (Fig. 2F, G). Furthermore, when compared to GSCs, normal neuron stem cells (Fig. S4B) and normal human astrocytes (Fig. S4C, D) also exhibited a decreased expression of ABCB4. In conclusion, our findings indicated that ABCB4 is specifically overexpressed in GSCs.

### ABCB4 contributes to the acquired resistance of TMZ in GSCs

**Fig. 3:**
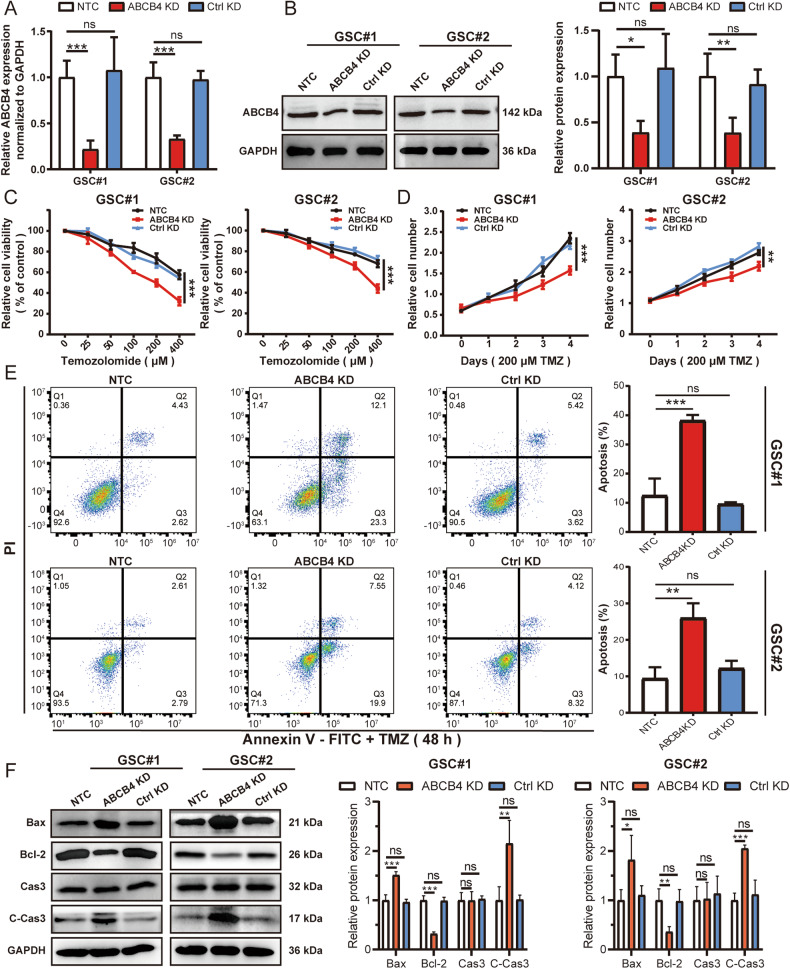
ABCB4 contributes to TMZ resistance in GSCs. **A** ABCB4 mRNA levels determined by qRT-PCR. **B** ABCB4 protein levels determined by WB. **C**, **D** Relative cell viability of NTC, ABCB4 KD, or Ctrl KD GSCs on TMZ treatment at indicated concentrations for 48 h or 200 µM for indicated times. **E** The apoptotic rates of GSC#1 or GSC#2 transfected with NTC, ABCB4 KD, or Ctrl KD upon TMZ (200 µM) for 48 h by flow cytometry. **F** WB analysis of apoptotic proteins in GSC#1 or GSC#2 transfected with NTC, ABCB4 KD, or Ctrl KD upon TMZ (200 µM) for 48 h. All experiments were repeated independently three times. Data are presented as mean ± standard deviation. *p < 0.05; **p < 0.01; ***p < 0.001; ns not significant.

To explore the role of ABCB4 in GSCs, stable ABCB4 knockdown (ABCB4 KD) GSCs were generated via lentiviral transduction. ABCB4 knockdown efficiency was validated by WB and qRT-PCR (Fig. 3A, B). CCK-8 results showed that, compared with the control group, the viability of ABCB4 KD GSCs was significantly decreased after TMZ treatment (Fig. 3C, D). Similarly, the results from the CFA analysis also demonstrated a significant reduction in the viability of ABCB4 KD GSCs after TMZ treatment, in comparison to the control group (Fig. S5A). The flow cytometry results also showed that the apoptosis rate of ABCB4 KD GSCs was significantly increased after TMZ treatment compared to that in the control group (Fig. 3E). The expression of apoptosis-related proteins including caspase-3, cleaved-caspase-3, Bax, and Bcl-2 determined using WB also suggested that, compared to the control group, the expression of Bax and cleaved-caspase-3 increased, while the expression of Bcl-2 decreased in ABCB4 KD GSCs treated with TMZ (Fig. 3F). At the same time, we examined the effect of ABCB4 itself on the proliferation of GSCs without TMZ treatment. The results demonstrate that ABCB4 had no significant effect on the proliferation of GSCs without TMZ intervention (Fig. S5A, B). Overall, these results suggested that ABCB4 is involved in the acquired drug resistance of GSCs to TMZ.

### Knockdown of ABCB4 enhances the chemosensitivity of GBM to TMZ therapy in vivo

**Fig. 4:**
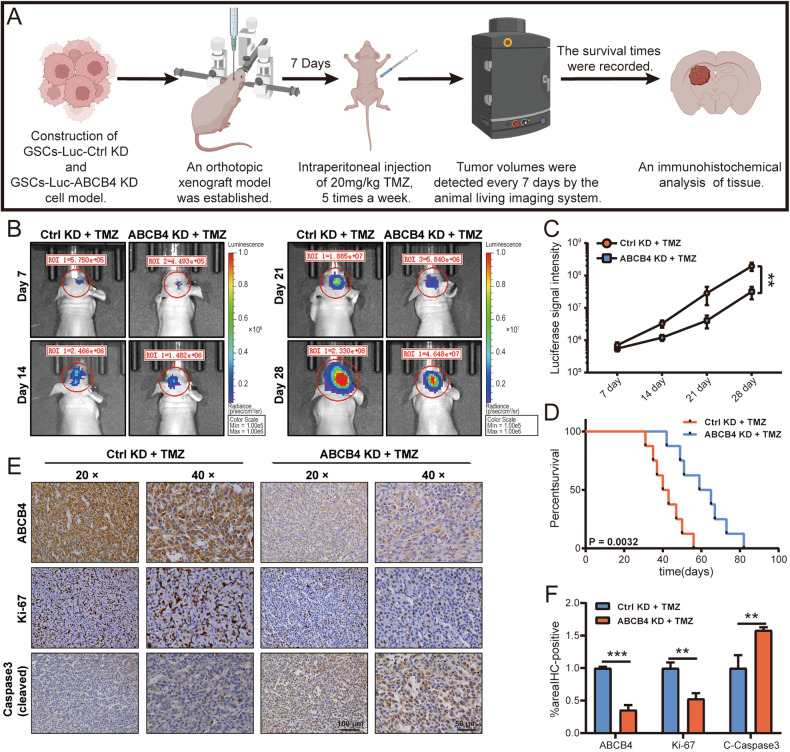
Knockdown of ABCB4 enhances the chemosensitivity of GBM to TMZ therapy in vivo. **A** Schematic of the in vivo model. **B** Bioluminescence imaging indicating tumour size over time. **C** Luminescent signal intensity of glioma-bearing mice in two groups. **D** Evaluation of animal survival carried out according to Kaplan–Meier analysis. **E** Immunohistochemical staining of ABCB4, Ki-67, and cleaved-caspase-3 in Ctrl KD group and ABCB4 KD group samples. Scale bar = 50/100 μm. **F** Quantification of ABCB4, Ki-67, and cleaved-caspase-3 intensity in immunohistochemical staining. Data are presented as mean ± standard deviation. *p < 0.05; **p < 0.01; ***p < 0.001; ns not significant.

To further investigate whether ABCB4 is involved in TMZ resistance in vivo, as shown in Fig. 4A, intracranial xenograft models of ABCB4 KD and its control group in nude mice were established. Bioluminescence imaging analysis shows ABCB4 knockdown significantly inhibited tumour growth (Fig. 4B, C). Moreover, the ABCB4 KD group exhibited longer survival than the control group (Fig. 4D). Xenografts knocking down ABCB4 significantly decreased Ki-67 levels and increased levels of cleaved-caspase-3 compared to control xenografts (Fig. 4E, F). These results indicated that ABCB4 knockdown enhanced chemosensitivity to TMZ in vivo. To verify whether ABCB4 itself can promote tumour growth, we took another group of nude mice to repeat the above experiment, but this time there was no TMZ treatment. Bioluminescence imaging analysis revealed that knockdown of the ABCB4 gene did not exert a significant impact on tumour growth (Fig. S5C, D). Furthermore, the survival rate of the ABCB4 knockdown group did not differ significantly from that of the control group (Fig. S5E). These findings indicate that ABCB4 itself does not play a substantial role in tumour growth.

### ABCB4-mediated resistance to TMZ is transmitted between cells

**Fig. 5:**
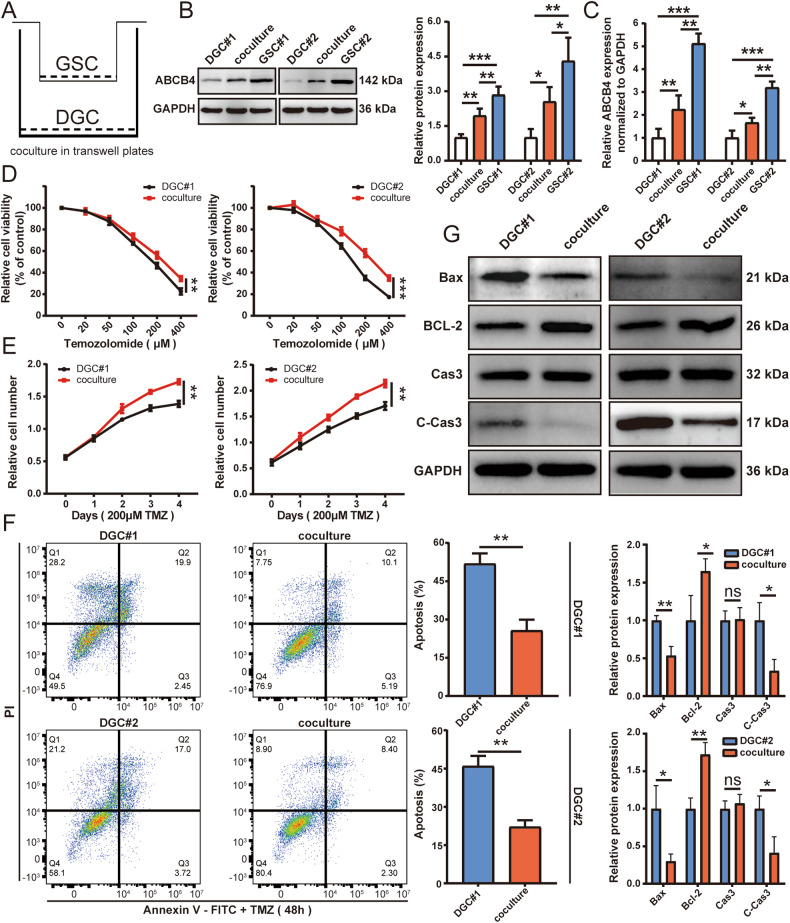
ABCB4-mediated resistance to TMZ is transmitted between cells. **A** Schematic of GSC–DGC co-culture. **B**. After 48 h of co-culture with GSCs, the ABCB4 protein content in DGCs was detected by WB. **C** After 48 h of co-culture with GSCs, the ABCB4 mRNA levels in DGCs was detected by qRT-PCR. **D**, **E** Relative cell viability of DGC or co-culture groups on TMZ treatment at indicated concentrations for 48 h or 200 µM for indicated times. **F** The apoptotic rates of DGC or co-culture groups upon TMZ (200 µM) for 48 h by flow cytometry. **G** WB analysis of apoptotic proteins in DGC or co-culture groups upon TMZ (200 µM) for 48 h. All experiments were repeated independently three times. Data are presented as mean ± standard deviation. *p < 0.05; **p < 0.01; ***p < 0.001; ns not significant.

To investigate whether ABCB4-mediated resistance to TMZ could be transmitted from GSCs to DGCs, co-culture experiments were performed (Fig. 5A) and changes in ABCB4 expression in DGCs detected by WB and qRT-PCR. ABCB4 expression in DGCs increased after co-culture with GSCs (Fig. 5B, C). Cell viability results showed that, compared with the DGC group, the viability of DGCs co-cultured with GSCs was significantly enhanced after TMZ treatment (Fig. 5D, E). Flow cytometry showed that the apoptosis rate of DGCs co-cultured with GSCs was significantly lower after TMZ treatment than that in the DGC group (Fig. 5F). The expression of apoptosis-related proteins including caspase-3, cleaved-caspase-3, Bax, and Bcl-2 determined using WB also suggested that compared to the DGC group, the expression of Bax and cleaved-caspase-3 decreased, while the expression of Bcl-2 increased in the co-cultured group after TMZ treatment (Fig. 5G). Taken together, these results suggest that the ABCB4-mediated resistance to TMZ can be transferred from GSCs to DGCs.

### Exosomes released by GSCs contain a high level of ABCB4

**Fig. 6:**
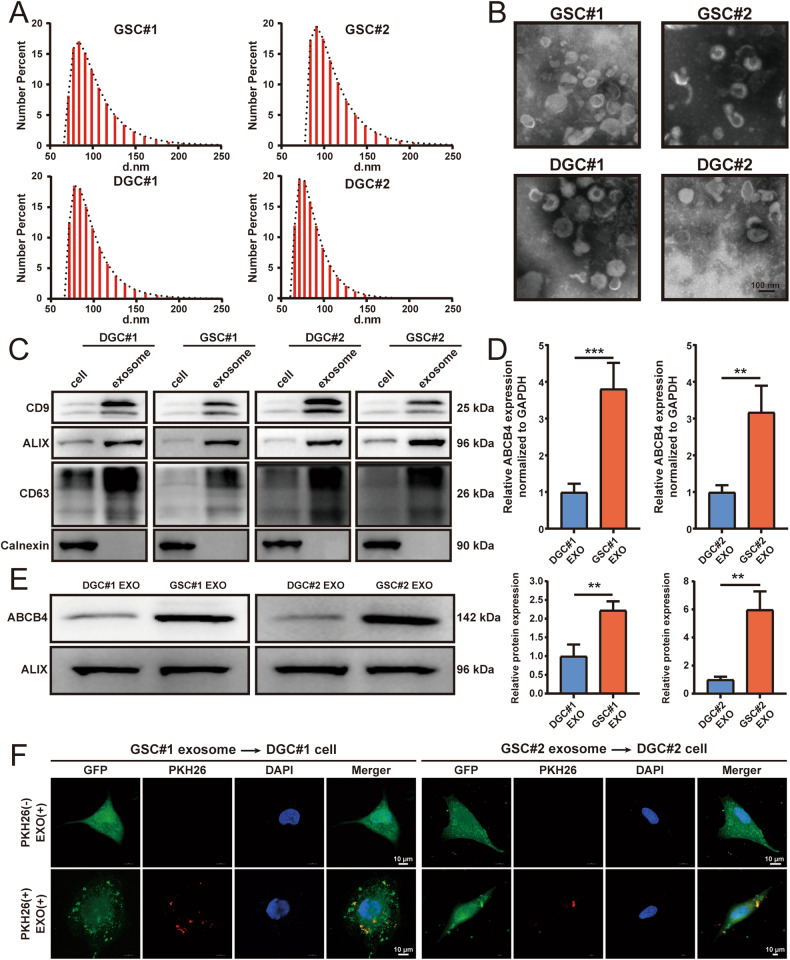
Exosomes released by GSCs contain a high level of ABCB4. **A** Size distribution of the isolated exosomes analysed by Zetasizer Nano-Zs. **B** Uranyl acetate negative stained TEM images of exosomes isolated from GSC#1, DGC#1, GSC#2, and DGC#2. Scale bar = 100 nm. **C** Immunoblotting for exosomal markers CD9, ALIX, and CD63, as well as negative control, calnexin. **D** Detection of ABCB4 relative expression levels in GSC and DGC derived exosomes by qRT-PCR. **E** Protein level of ABCB4 in exosomes derived from GSC and DGC detected by WB. **F** Exosomes secreted by GSCs were labelled with PKH26 and incubated with DGCs for 24 h. Representative laser confocal images showing exosomes merged with DGC#1 (left panel) and DGC#2 (right panel). Exosomes are labelled with PKH26 (red), and DGCs are labelled with GFP (green). Nuclei stained with DAPI (blue). Scale bar = 10 μm. All experiments were repeated independently three times. Data are presented as mean ± standard deviation. *p < 0.05; **p < 0.01; ***p < 0.001; ns not significant.

We investigated whether the ABCB4-mediated transfer of TMZ resistance from GSCs to DGCs is mediated by exosomes. First, exosomes were collected from GSC#1, DGC#1, GSC#2, and DGC#2 cell culture media by ultracentrifugation, and exosome size examined using a Zetasizer Nano-Zs analyser, which showed that the diameter of most particles was within the typical exosomal range (40–150 nm, Fig. 6A). GSC#1, DGC#1, GSC#2, and DGC#2 exosome morphology was analysed by TEM, which revealed that they were round and oval in shape, with a diameter of 40–150 nm (Fig. 6B). The expression of the known exosomal markers, CD9, ALIX, and CD63, as well as that of the endoplasmic reticulum protein, calnexin, was compared by WB between exosome extracts and donor cells. CD9, ALIX, and CD63 were enriched in the exosome fractions compared to the corresponding donor cells, whereas calnexin was exclusively expressed in the donor cells (Fig. 6C). Thus, we concluded that this procedure led to the isolation of exosomes from GSC#1, DGC#1, GSC#2, and DGC#2. qRT-PCR and WB analysis of ABCB4 expression in GSC#1, DGC#1, GSC#2, and DGC#2 exosomes showed that ABCB4 was significantly enriched in GSC exosomes compared to DGC exosomes (Fig. 6D, E). To detect the ability of the DGC#1/DGC#2 cells to internalise exosomes derived from GSC#1/GSC#2 cells. The exosome was labelled with red fluorescent dye PKH26, and the exosome (10 μg/ml) was co-cultured with DGCs for 24 h, and then fluorescence analysis was performed by a laser confocal microscope. The results showed that the exosomes were internalised by the DGC#1/DGC#2 cells and distributed around the nucleus (Fig. 6F). These data demonstrate that ABCB4 is enriched in GSC-derived exosomes and that GSC exosomes can enter DGCs.

### ABCB4-mediated resistance to TMZ is transmitted from cell to cell through exosomes

**Fig. 7:**
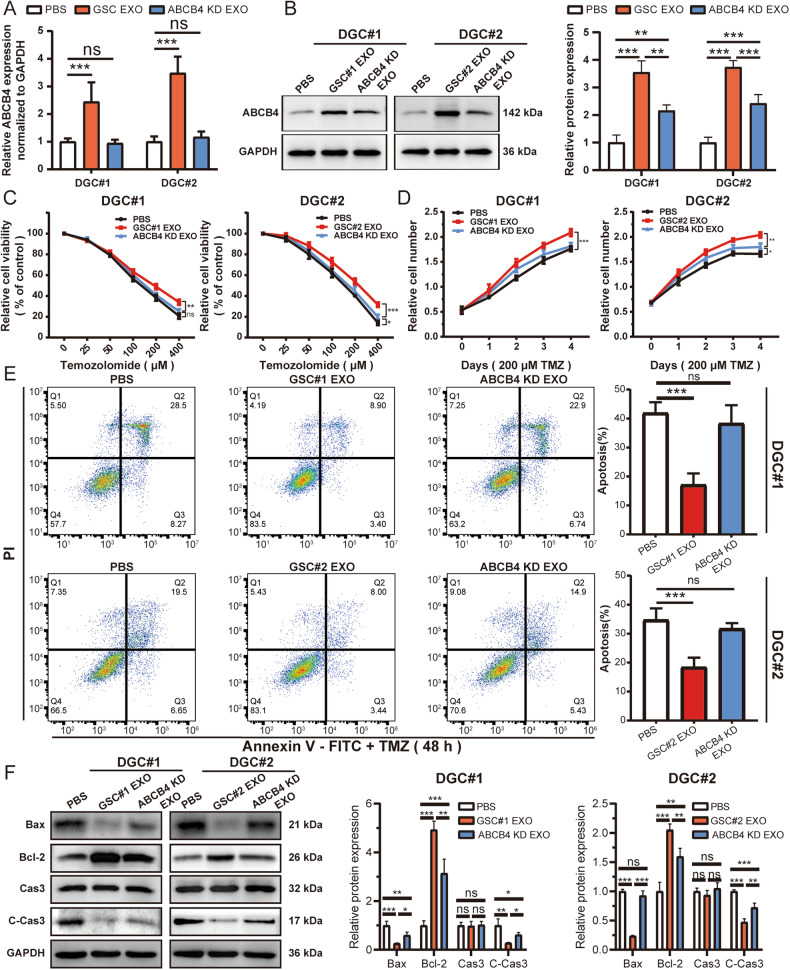
ABCB4-mediated TMZ resistance is transmitted from GSCs to DGCs via exosomes. **A** mRNA expression of ABCB4 in DGCs treated with exosomes from GSCs or ABCB4 KD GSCs detected by qRT-PCR. **B** Protein expression of ABCB4 in DGCs cells treated with exosomes from GSCs or ABCB4 KD GSCs detected by WB. **C**, **D** Relative cell viability of DGCs of different exosome treatment groups on TMZ treatment at indicated concentrations for 48 h or 200 µM for indicated times. **E** The apoptotic rates of DGCs of different exosome treatment groups upon TMZ (200 µM) for 48 h by flow cytometry. **F** WB analysis of apoptotic proteins in DGCs of different exosome treatment groups upon TMZ (200 µM) for 48 h. All experiments were repeated independently three times. Data are presented as mean ± standard deviation. *p < 0.05; **p < 0.01; ***p < 0.001; ns not significant.

To determine whether ABCB4 could be transferred from GSC to DGC cells via exosomes, exosomes from GSC (GSC EXO) and ABCB4 KD GSC (ABCB4 KD EXO) culture media were collected by ultracentrifugation. Next, following the quantification of exosome concentration using the BCA method, cells were then subjected to treatment with either PBS or 10 μg/ml exosomes in accordance with their respective treatment groups. The expression of ABCB4 in DGC cells treated with exosomes from GSCs or ABCB4 KD GSCs, detected by qRT-PCR and WB, showed that the content of ABCB4 in DGCs treated with GSC EXO was higher than that in the PBS control group (Fig. 7A, B). Compared to the PBS control group, the content of ABCB4 in DGCs treated with ABCB4 KD EXO increased slightly, but it was not as significant as that in the GSC EXO group (Fig. 7A, B). Cell viability results showed that, compared with the PBS control group, the viability of DGCs treated with GSC EXO was significantly enhanced after TMZ treatment (Fig. 7C, D). However, the viability of DGCs treated with ABCB4 KD EXO increased slightly, but not as significantly as that of the GSC EXO group (Fig. 7C, D). The apoptotic rate of cells treated with TMZ detected by flow cytometry showed the same result (Fig. 7E). The expression of apoptosis-related proteins including caspase-3, cleaved-caspase-3, Bax, and Bcl-2 determined by WB also suggested that compared to the PBS control group, the expression of Bax and cleaved-caspase-3 decreased, while the expression of Bcl-2 increased in the GSC EXO group after TMZ treatment (Fig. 7F). However, compared to the PBS control group, after TMZ treatment, the apoptosis markers of DGCs treated with ABCB4 KD EXO changed slightly, but it was not as significant as that of GSC EXO group (Fig. 7F). In summary, ABCB4-mediated TMZ resistance was transmitted from GSCs to DGCs via exosomes.

### ATF3 positively regulates the expression of ABCB4

**Fig. 8:**
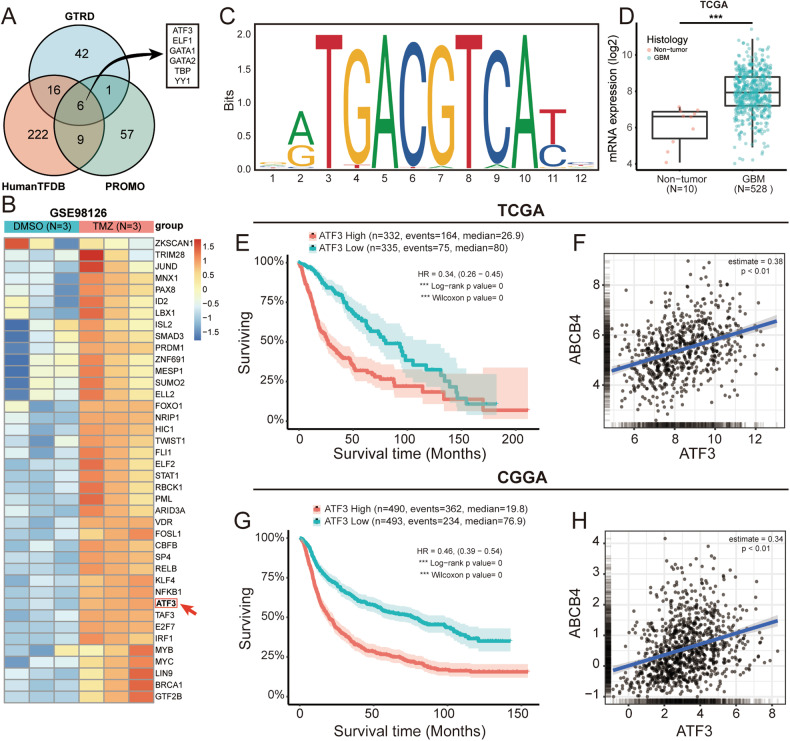
ATF3 may positively regulate ABCB4 expression. **A** Venn diagrams indicate the transcription factors that can bind to the ABCB4 promoter in the GTRD, PROMO, and Human TFDB databases. **B** Heat maps show increased expression of transcription factors in GSCs after TMZ treatment. **C** Sequence motif calculated to show the ATF3 binding motif through the JASPAR database. **D** The box plot in TCGA database shows the expression of ATF3 between normal tissue (red) and GBM (green). **E** Prognosis efficiency of ATF3 in WHO grade patients within TCGA RNA-seq data. **F** Correlation between gene expression of ABCB4 and ATF3 in the TCGA dataset. **G** Prognosis efficiency of ATF3 in WHO grade patients within the CGGA RNA-seq data. **H** Correlation between gene expression of ABCB4 and ATF3 in the CGGA dataset. *p < 0.05; **p < 0.01; ***p < 0.001; ns not significant.

To explore the mechanisms that promote ABCB4 upregulation in GSCs, we focused on the changes in transcription factor regulation. First, transcription factors that could bind to the ABCB4 promoter were predicted using the Gene Transcription Regulation Database [33] (GTRD, http://gtrd.biouml.org/), PROMO [34] (http://alggen.lsi.upc.es/cgi-bin/promo_v3/promo/promoinit.cgi?dirDB=TF_8.3/), and Human Transcription Factor Database [35] (Human TFDB, http://bioinfo.life.hust.edu.cn/HumanTFDB#!/). By examining the intersection of GTRD, PROMO, and Human TFDB, we obtained six transcription factors, including ATF3 (Fig. 8A). The DESeq2 package in R was used to analyse the GSE98126 dataset, and the expression of several transcription factors, including ATF3, increased in the GSCs treated with TMZ (Fig. 8B). The JASPAR database [36] indicated that ATF3 binds to the ABCB4 promoter (Fig. 8C). Therefore, we focused on ATF3 in this study. To explore whether ATF3 promotes the progression of GBM, as well as ABCB4, we analysed its expression in TCGA and CGGA. First, the data from TCGA showed that ATF3 was highly expressed in GBM tissues compared to normal tissues (Fig. 8D). Concurrently, the TCGA (Fig. 8E) and CGGA (Fig. 8G) data suggested that high ATF3 expression was associated with poor prognosis. We then used TCGA and CGGA to analyse the correlation between ABCB4 and ATF3. These findings indicated a positive correlation between ABCB4 and ATF3 (Fig. 8F, H). In conclusion, ATF3 may positively regulate ABCB4 expression.

### ABCB4 is a direct transcriptional target of ATF3

**Fig. 9:**
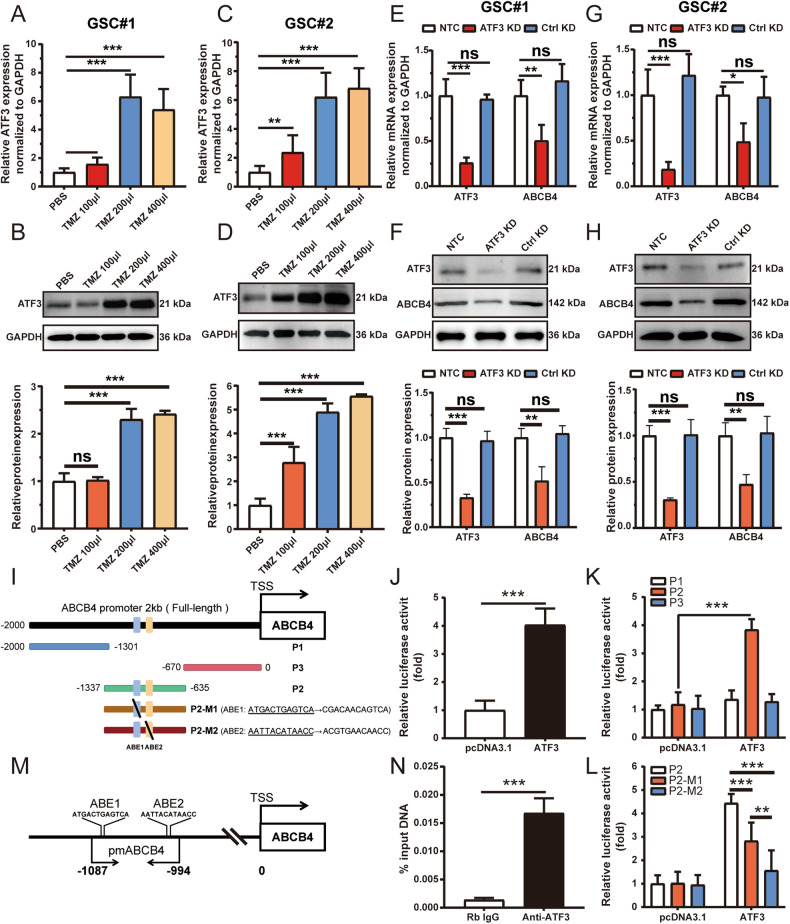
ABCB4 is a direct transcriptional target of ATF3. **A** mRNA level of ATF3 in GSC#1 measured by qRT-PCR. **B** Protein level of ATF3 in GSC#1 measured by WB. **C** mRNA level of ATF3 in GSC#2 measured by qRT-PCR. **D** Protein level of ATF3 in GSC#2 measured by WB. **E** Levels of ATF3 and ABCB4 mRNA in GSC#1 measured by qRT-PCR after ATF3 knockdown. **F** Levels of ATF3 and ABCB4 proteins in GSC#1 measured by WB after ATF3 knockdown. **G** Levels of ATF3 and ABCB4 mRNA in GSC#2 measured by qRT-PCR after ATF3 knockdown. **H** Levels of ATF3 and ABCB4 proteins in GSC#2 measured by WB after ATF3 knockdown. **I** Diagram showing the relative positions of full-length (FL) and fragments of ABCA4 promoter reporters. **J** Responses of the FL reporter of ABCA4 promoter to ATF3. **K** Responses of the individual fragments of ABCA4 promoter to ATF3. **L** Reporter assays of the P2 fragment of the ABCA4 promoter containing a mutated ABE. **M** Schematic illustrating the relative positions of qPCR probes to putative ABEs for ChIP-qPCR experiments. **N** Antibody-pulled down chromatins analysed by qPCR. All experiments were repeated independently three times. Data are presented as mean ± standard deviation. *p < 0.05; **p < 0.01; ***p < 0.001; ns not significant.

Cell experiments were conducted to verify the bioinformatics analysis results. First, qRT-PCR and WB showed that the expression of ATF3 in GSC#1 (Fig. 9A, B) and GSC#2 (Fig. 9C, D) increased with increasing TMZ concentrations. We examined the effect of ATF3 knockdown on ABCB4 expression. The results showed that the expression of ABCB4 in GSC#1 (Fig. 9E, F) and GCS#2 (Fig. 9G, H) decreased following ATF3 knockdown. To investigate whether ATF3 directly interacted with the ABCB4 promoter element, we cloned the full-length (FL) ABCB4 promoter (2 kb, Fig. 9I) into a luciferase reporter plasmid. The 2 kb-FL reporter was then co-transfected with pcDNA3.1 empty vector or ATF3-containing plasmids into GSC#1, and the resultant relative light units demonstrated a robust and positive response induced only by ATF3 (Fig. 9J). The FL reporter was fragmented into three overlapping segments (P1, P2, and P3), with P2 containing two ATF3 binding elements (ABEs), ATGACTGAGTCA, and AATTACATAACC (Fig. 9I). The luciferase reporter data were consistent with the P2 fragment directly interacting with ATF3 (Fig. 9K). Next, we mutated ABE1 (P2-M1) and ABE2 (P2-M2) in the P2 fragment (Fig. 9I) and found that the luciferase activity of P2-M1 and P2-M2 was lower than that of P2 (Fig. 9L). To substantiate the putative interaction between the P2 fragment and ATF3, we performed ChIP-qPCR to probe the genomic occupancy of ATF3 at the P2 sequence (Fig. 9M). The results of the ChIP assay further verified that ATF3 bound tightly to the P2 fragment of ABCB4 (Fig. 9N). Taken together, these data suggested that ABCB4 is a direct transcriptional target of ATF3.

## Discussion

GBM is the most common and fatal primary tumour in the central nervous system [37]. TMZ chemotherapy is recognised as the gold standard treatment for GBM [37]; however, the emergence of acquired drug resistance has become an important obstacle to their successful treatment [38]. Therefore, improving chemosensitivity to TMZ is key to improving its therapeutic effects [39]. Recent studies have revealed a strong association between GSCs and drug resistance [40], and GSCs are the main cause of GBM resistance to chemotherapy drugs, such as TMZ, which significantly reduces the effectiveness of treatment [41]. Concurrently, some studies have found that the inhibitory effect of TMZ on GSCs at conventional chemotherapy doses is relatively weak, far less significant than that on common glioma cells, further suggesting that the GSC drug resistance mechanism may be significantly different from that of common glioma cells [42]. Therefore, to develop effective anti-drug resistance strategies, further study of the molecular mechanisms underlying drug resistance in GSCs is required.

Current studies suggest that GSCs may develop resistance to TMZ treatment through a multidrug resistance mechanism in which the ABC transporter superfamily plays a key role [43]. The ABC transporter superfamily is one of the largest membrane protein families found in various organisms [20]. They are also called efflux pumps because they transport various endogenous and exogenous molecules across membranes using the energy generated by ATP hydrolysis [44]. ABC transporters have been found to mediate the efflux of a variety of chemotherapeutics in different tumour structures, thereby reducing the effectiveness of chemotherapy [45]. Therefore, ABC transporters are closely associated with multidrug resistance in tumours [18]. Several members of the ABC transporter superfamily are significantly overexpressed in CD133-positive GSCs [20]. In addition, overexpression of ABCG2 in GSCs is associated with high resistance of GSCs to chemotherapeutic agents [21]. These results suggest that the ABC transporter superfamily may be involved in the development of drug resistance in GSCs; however, the specific mechanism remains unclear.

Therefore, this study focused on the mechanism by which GSCs participate in TMZ resistance through the ABC transporter superfamily. First, we analysed changes in ABC transporter expression in GSCs before and after TMZ treatment using the GEO database [46] and found that several ABC transporters, including ABCB4, were significantly upregulated in TMZ-treated GSCs. Further bioinformatics analysis showed that only a few ABC transporters had a significant impact on the prognosis, of which ABCB4 had the greatest impact, so we chose ABCB4 to study. Next, using a biological database and clinical glioma samples, we found that ABCB4 is highly expressed in GBM, and that ABCB4 overexpression is highly correlated with poor prognosis in patients with GBM. CGGA database analysis revealed a positive correlation between ABCB4 expression and GSC markers CD133, Nestin, and OCT-4. Confocal microscopy also revealed that ABCB4 and CD133 were co-localised in GBM tissues, which supported the view that GSCs express ABCB4. ABCB4 expression was also found in human GSCs isolated and cultured from GBM patient samples, and was significantly higher in GSCs than in DGCs. In conclusion, our findings suggested that ABCB4 is specifically overexpressed in GSCs. We knocked down ABCB4 expression in GSCs using a lentivirus. In vitro and in vivo experiments showed that ABCB4 knockdown significantly increased the chemosensitivity of GSCs to TMZ. Additionally, ABCB4 is involved in acquired resistance of GSCs to TMZ. Interestingly, GEO database analysis showed that TMZ reduced ABCB1 expression in GSCs. ABCB1 is structurally similar to ABCB4 and generally thought to be associated with drug resistance [47]; however, previous studies have suggested that ABCB4 does not confer multidrug resistance [48]. Nevertheless, the study by Fischer et al. overturned this view and found that ABCB4 could participate in cell resistance to chemicals in the absence or with low expression of ABCB1 [23]. This coincides with our results; that is, after TMZ treatment of GSCs, the expression of ABCB1 decreased, while the expression of ABCB4 increased, and the increased expression of ABCB4 was involved in the regulation of drug resistance of GSCs to TMZ.

Recently, the increase in the study of the tumour microenvironment has provided a new concept for tumour drug resistance research [49]. Cell-to-cell communication plays a crucial role in the tumour microenvironment [50]. Exosomes secreted by tumour cells, as key mediators of cell-mediated communication, provide a new entry point for the study of malignant tumour phenotypes and drug resistance and have become a new hot spot in the field of tumour research [51]. Exosomes are currently the most clearly defined vesicles, which are formed by intracellular multi-vesicular bodies (MVBs), fused with cell membranes and secreted to the outside [25]. Exosomes transport their contents such as proteins, lipids, and nucleic acids directly into receptor cells to exert biological regulatory activities [52]. Increasing evidence shows that exosomes are not only related to tumour cell proliferation, epithelial-mesenchymal transformation, and invasion, but also play an important role in the drug resistance of tumour cells [28, 53, 54]. Therefore, exosomes secreted by tumour cells provide a new model for studying drug resistance in tumours. Recent studies have suggested that exosomes mediate tumour drug resistance by transferring ABC transporters between cells [28]. Sequencing of exosomes secreted by cancer cells has revealed the presence of ABCB4, suggesting that ABCB4 may transmit drug resistance between cells through exosomes [29]. However, the specific mechanisms remain unclear and require further investigation.

Our results indicate that when GSCs were co-cultured with DGCs, ABCB4 expression in DGCs was significantly increased, and resistance to TMZ was correspondingly enhanced. Taken together, these results suggest that the ABCB4-mediated resistance to TMZ can be transferred from GSCs to DGCs. Subsequently, we extracted and identified the exosomes secreted by GSCs and DGCs and found that the exosomes secreted by GSCs contained more ABCB4 than those secreted by DGCs. Next, DGCs were treated with exosomes secreted by GSCs, and the chemoresistance of DGCs to TMZ was significantly increased. However, when DGCs were treated with ABCB4 KD EXO, their chemoresistance to TMZ did not change significantly. These results revealed that ABCB4-mediated resistance to TMZ can be transmitted via exosomes.

Finally, to investigate the mechanism of ABCB4 upregulation in GSCs after TMZ treatment, we focused on the changes in transcription factor regulation. First, using a transcription factor database, we identified six transcription factors, including ATF3. Subsequently, analysis of the GSE98126 dataset using R language revealed that only ATF3 expression increased in TMZ-treated GSCs. In addition, the JASPAR database predicted that ATF3 regulates ABCB4 transcription. Therefore, we focused on ATF3. Our results suggest that ATF3 is highly expressed in GBM and is negatively correlated with poor prognosis. ATF3 expression positively correlated with ABCB4 expression, and ABCB4 expression was affected by ATF3. In conclusion, ATF3 positively regulates ABCB4 expression. Finally, we confirmed ABCB4 as a direct transcriptional target of ATF3 using luciferase and ChIP-qPCR assays. This finding corroborates the study by Xu et al., who found that ATF3 upregulates the expression of UPF1 and Nestin, maintaining the stemness of GSCs [55]. Our experiments also suggested that ATF3 may promote drug resistance in GSCs by upregulating ABCB4 expression.

**Fig. 10:**
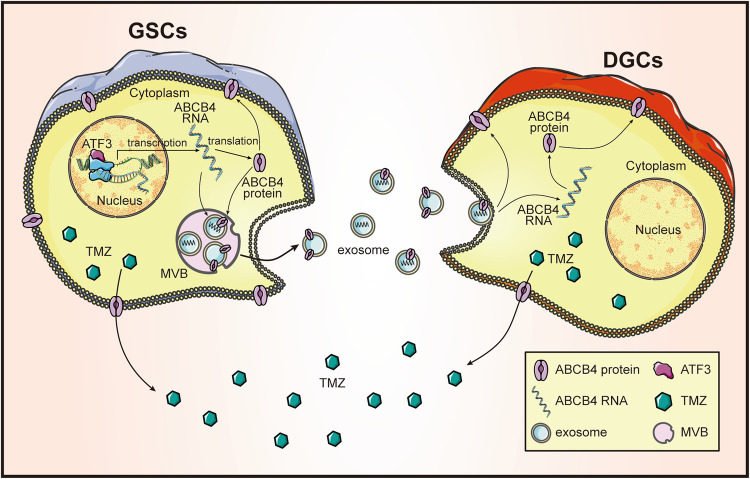
Mechanism diagram. ATF3 promotes transcription of ABCB4 in GSCs, leading to ABCB4 overexpression, thereby promoting resistance of GSCs to TMZ. Concurrently, GSCs transfer ABCB4 to DGCs via exosomes, so that DGCs also increase resistance to TMZ. MVB multivesicular body.

In conclusion, we found that ABCB4 is highly expressed in GSCs. Moreover, high expression of ABCB4 promoted the resistance of GSCs to TMZ. We also found that ABCB4-mediated resistance to TMZ was transmitted between cells. Our study found that the transmission of this type of drug resistance is carried out through exosomes secreted by GSCs, that is, GSCs secrete exosomes containing ABCB4 that enter DGCs, resulting in high expression of ABCB4 in DGCs, thus promoting DGCs to become resistant to TMZ. We also found that ABCB4 overexpression in GSCs was mediated by the transcription factor ATF3. Therefore, we propose that ATF3 promotes ABCB4 transcription in GSCs, resulting in ABCB4 overexpression, thereby promoting GSC resistance to TMZ. Simultaneously, GSCs deliver ABCB4 to DGCs via exosomes, also increasing their resistance to TMZ. The specific mechanism is illustrated in Fig. 10. These findings contribute to a deeper understanding of the mechanisms underlying drug resistance in GBM and provide novel insights into its treatment.

### Supplementary information

Supplementary figures and legends
