# Supplementary material for: Glioblastoma stem cells deliver ABCB4 transcribed by ATF3 via exosomes conferring glioblastoma resistance to temozolomide
Source: Cell Death Dis. 2024 May 6;15(5):318. doi: 10.1038/s41419-024-06695-6 (PMC11074105; doi:10.1038/s41419-024-06695-6)
Supplement: Supplementary file 1 — Supplementary figures and legends [file 41419_2024_6695_MOESM1_ESM.docx]

**Supplementary Figures**


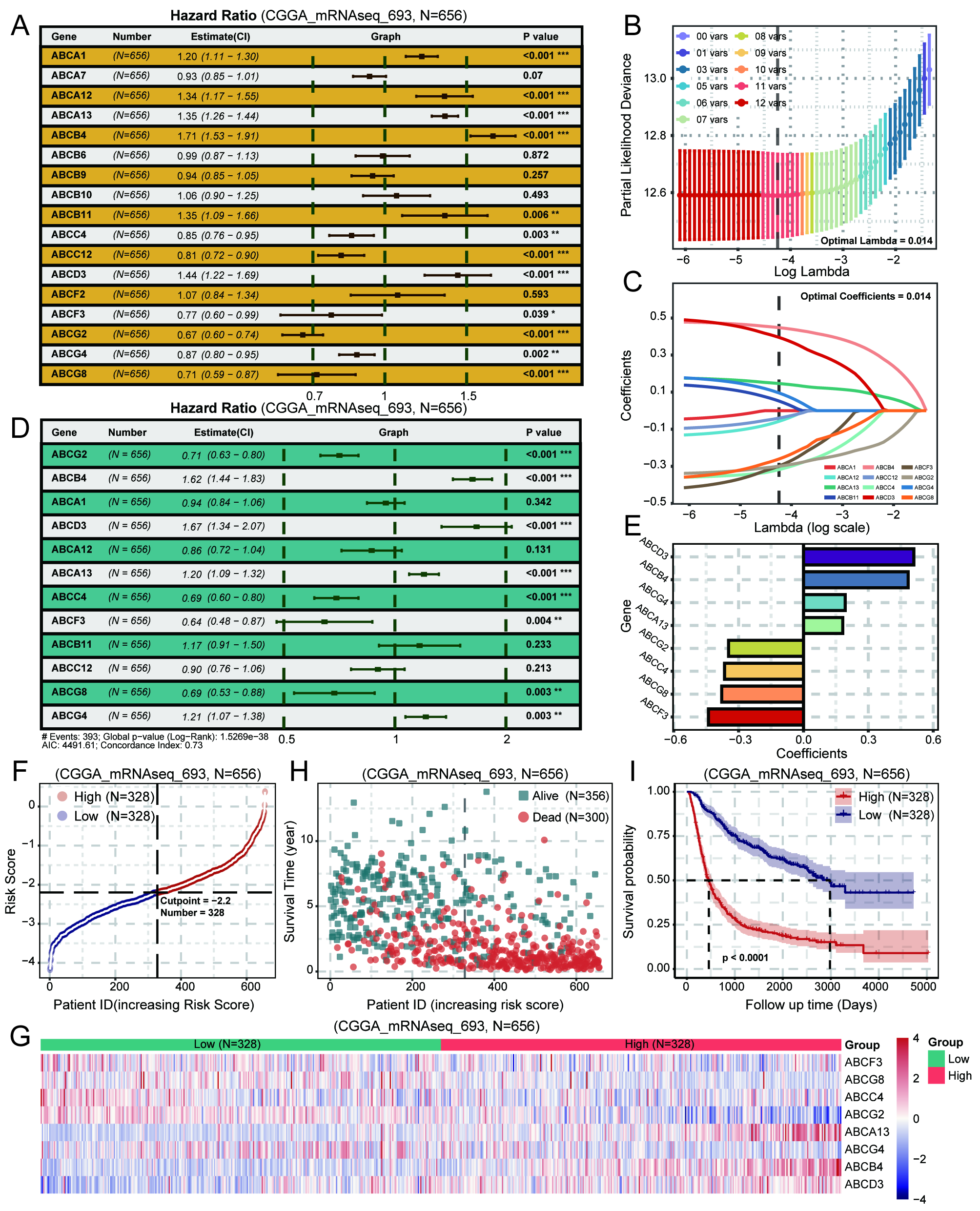


**Fig. S1 Prognostic model construction based on ABC transporter gene expression in the CGGA mRNA_693 dataset. A.** Forest plot of univariate Cox proportional hazards regression analysis for the upregulated members of the ABC transporter family (CGGA_mRNAseq_693, N = 656). **B.** 10-fold cross-validation used to determine the optimal λ value. **C.** Coefficient profiles of the LASSO model. **D.** Forest plot of multivariate Cox proportional hazards regression analysis of the best members from the LASSO model (CGGA_mRNAseq_693, N = 656). **E.** Variables identified by the multivariate Cox model and their corresponding coefficients. **F.** Stratification into high-risk (N = 328) and low-risk (N = 328) groups based on the median overall risk score. **G.** Expression profiles of individual variables in the model across high-risk (N = 328) and low-risk (N = 328) groups. **H.** Distribution plot of survival status (Group Alive = 356, Group Dead = 300). **I.** Kaplan–Meier survival curves between the high-risk (N = 328) and low-risk (N = 328) groups.


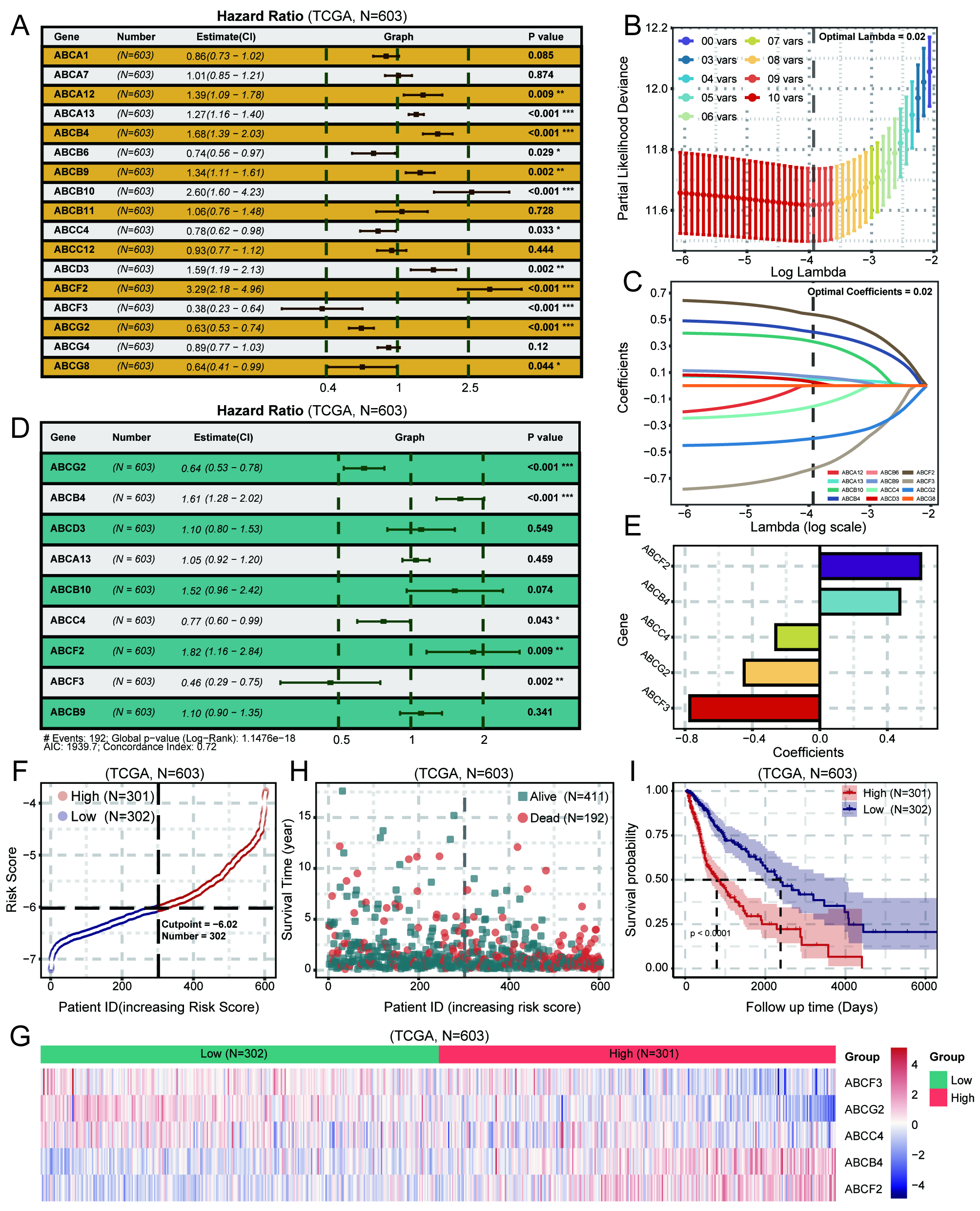


**Fig. S2 Prognostic model construction based on ABC transporter gene expression in TCGA dataset. A.** Forest plot of univariate Cox proportional hazards regression analysis for the upregulated members of the ABC transporter family (TCGA, N = 603). **B.** 10-fold cross-validation used to determine the optimal λ value. **C.** Coefficient profiles of the LASSO model. **D.** Forest plot of multivariate Cox proportional hazards regression analysis of the best members from the LASSO model (TCGA, N = 603). **E.** Variables identified by the multivariate Cox model and their corresponding coefficients. **F.** Stratification into high-risk (N = 302) and low-risk (N = 301) groups based on the median overall risk score. **G.** Expression profiles of individual variables in the model across high-risk (N = 302) and low-risk (N = 301) groups. **H.** Distribution plot of survival status (Group Alive = 411, Group Dead = 192). **I.** Kaplan–Meier survival curves between the high-risk (N = 302) and low-risk (N = 301) groups.


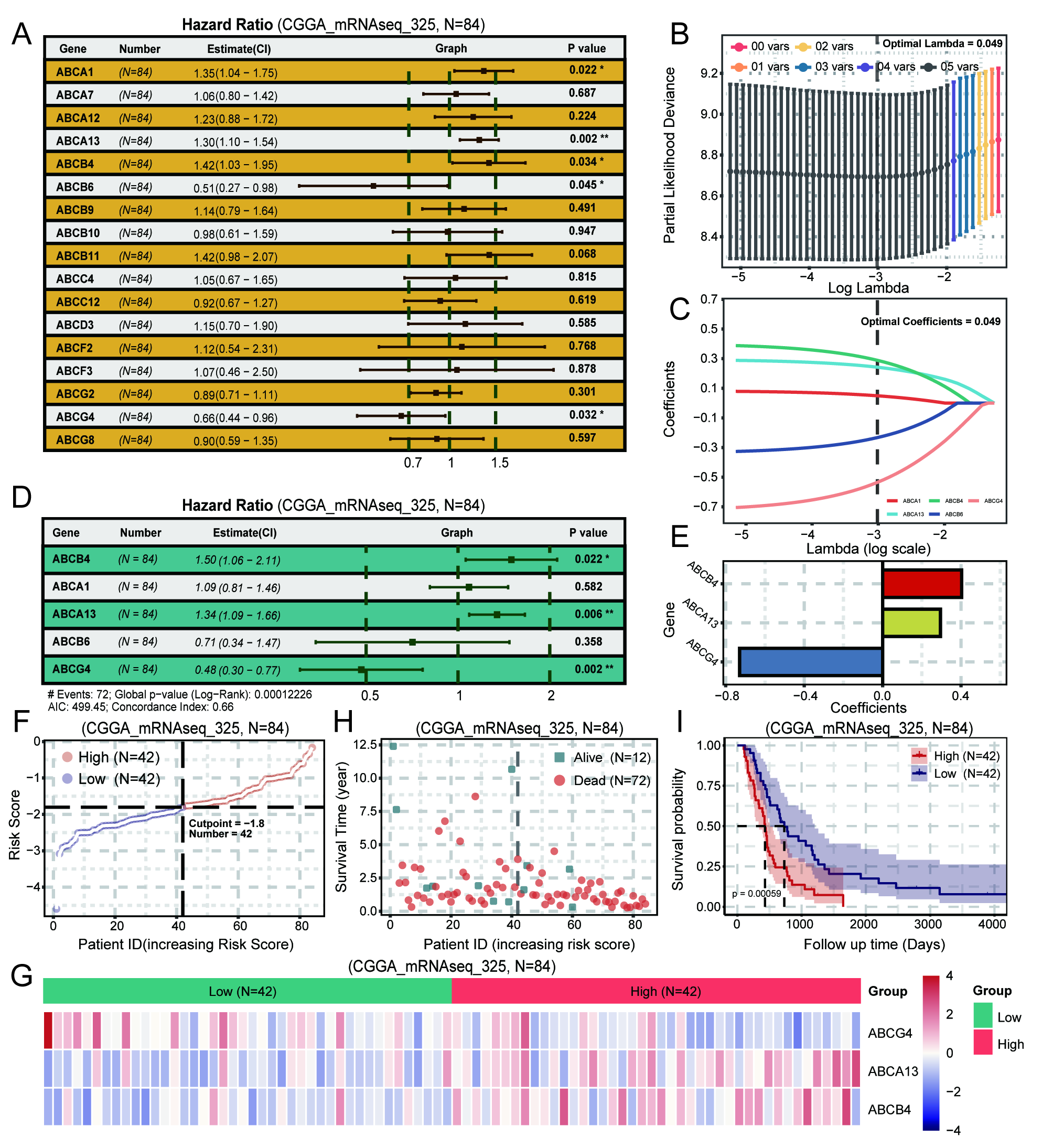


**Fig. S3 Prognostic model construction for GBM samples (N = 84) based on ABC transporter gene expression in the CGGA mRNA_325 dataset. A.** Forest plot of univariate Cox proportional hazards regression analysis for the upregulated members of the ABC transporter family (CGGA_mRNA_325, N = 84). **B.**10-fold cross-validation used to determine the optimal λ value. **C.** LASSO coefficient profiles of the prognosis-related DEGs. **D.** Forest plot of multivariate Cox proportional hazards regression analysis of the selected members from the LASSO model (CGGA_mRNA_325, N = 84). **E.** Variables identified by the multivariate Cox model and their corresponding coefficients. **F.** Stratification into high-risk (N = 42) and low-risk (N = 42) groups based on the median overall risk score. **G.** Expression profiles of individual variables in the model across high-risk (N = 42) and low-risk (N = 42) groups. **H.** Distribution plot of survival status (Group Alive = 12, Group Dead = 72). **I.** Kaplan-Meier survival curves between the high-risk (N = 42) and low-risk (N = 42) groups.


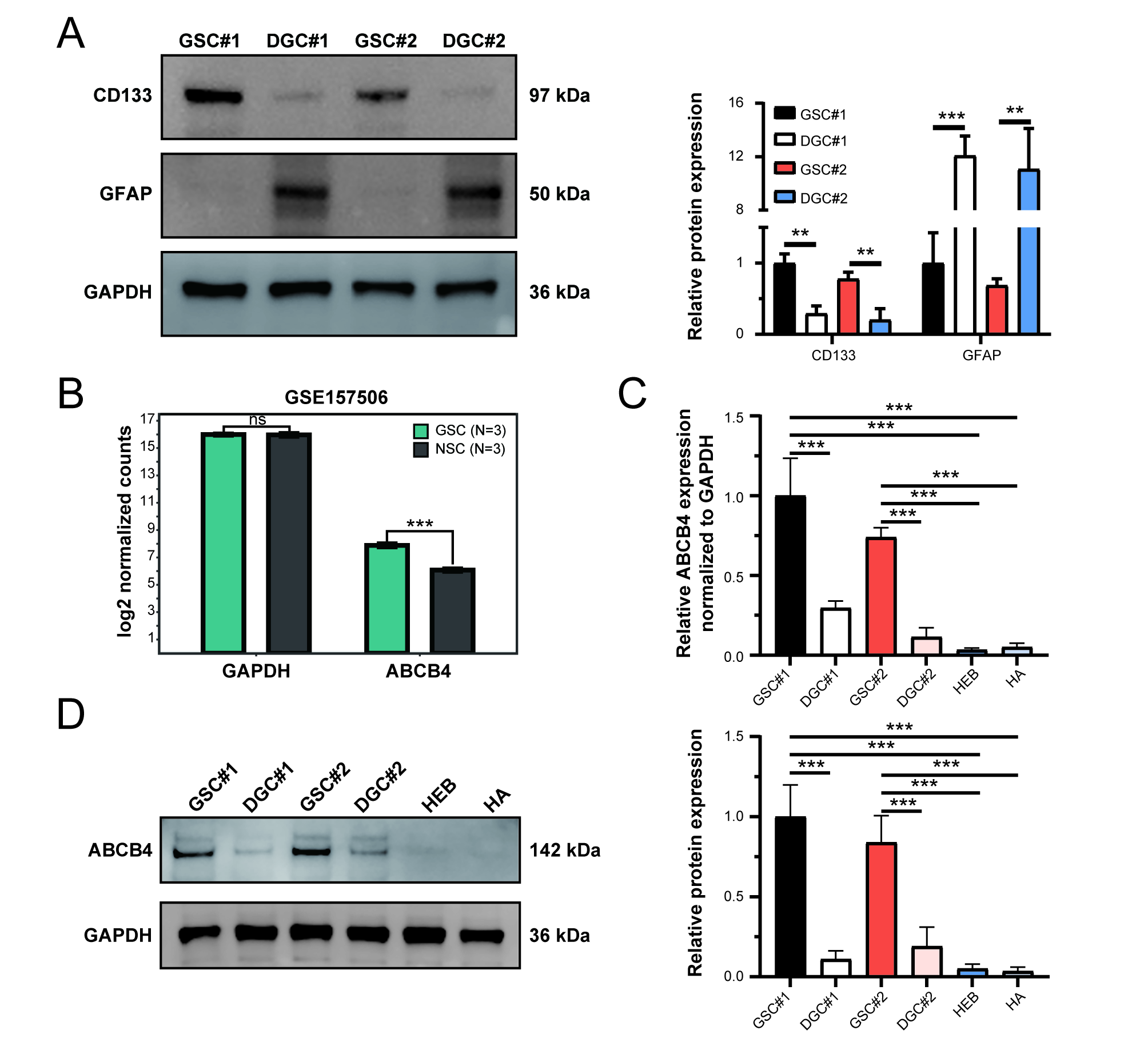


**Fig. S4 Low expression of ABCB4 in normal neural stem cells (NSC) and normal human astrocytes (HA). A.** Detection of CD133 and GFAP protein levels in GSCs and DGCs via WB. **B.** The GSE157506 dataset shows ABCB4 expression in GSC (N = 3) and NSC (N = 3). **C.** ABCB4 mRNA levels in different cells were detected by qRT-PCR. **D.** ABCB4 protein levels in different cells were detected by WB.


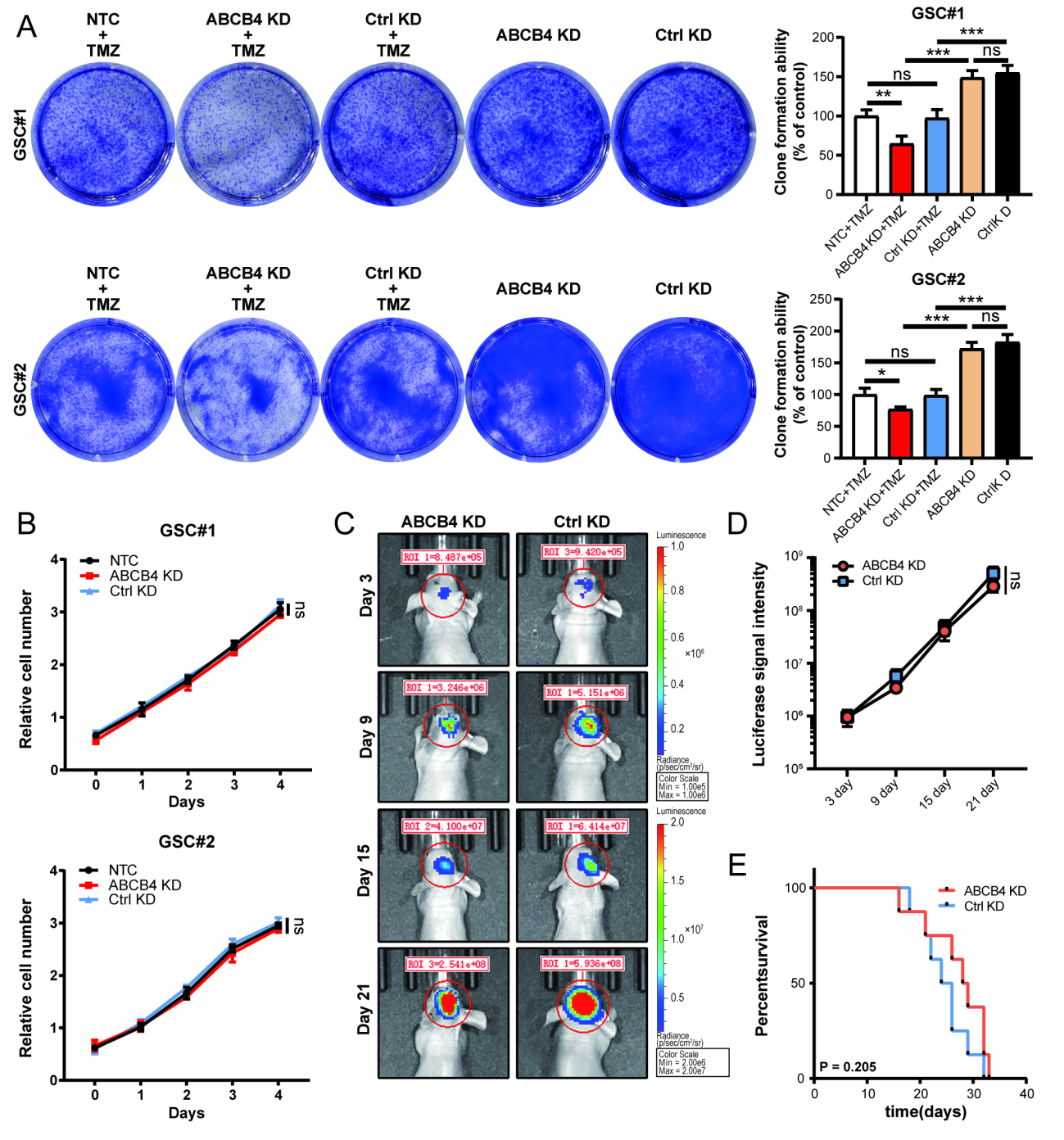


**Fig. S5 ABCB4 itself did not affect the tumour growth in the absence of TMZ.** **A.** The growth of GSCs was determined by a plate colony formation assay. **B.** The growth of GSCs was examined by CCK-8. **C.** Bioluminescence imaging indicating tumour size over time. **D.** Luminescent signal intensity of glioma-bearing mice in two groups. **E.** Evaluation of animal survival carried out according to Kaplan–Meier analysis.


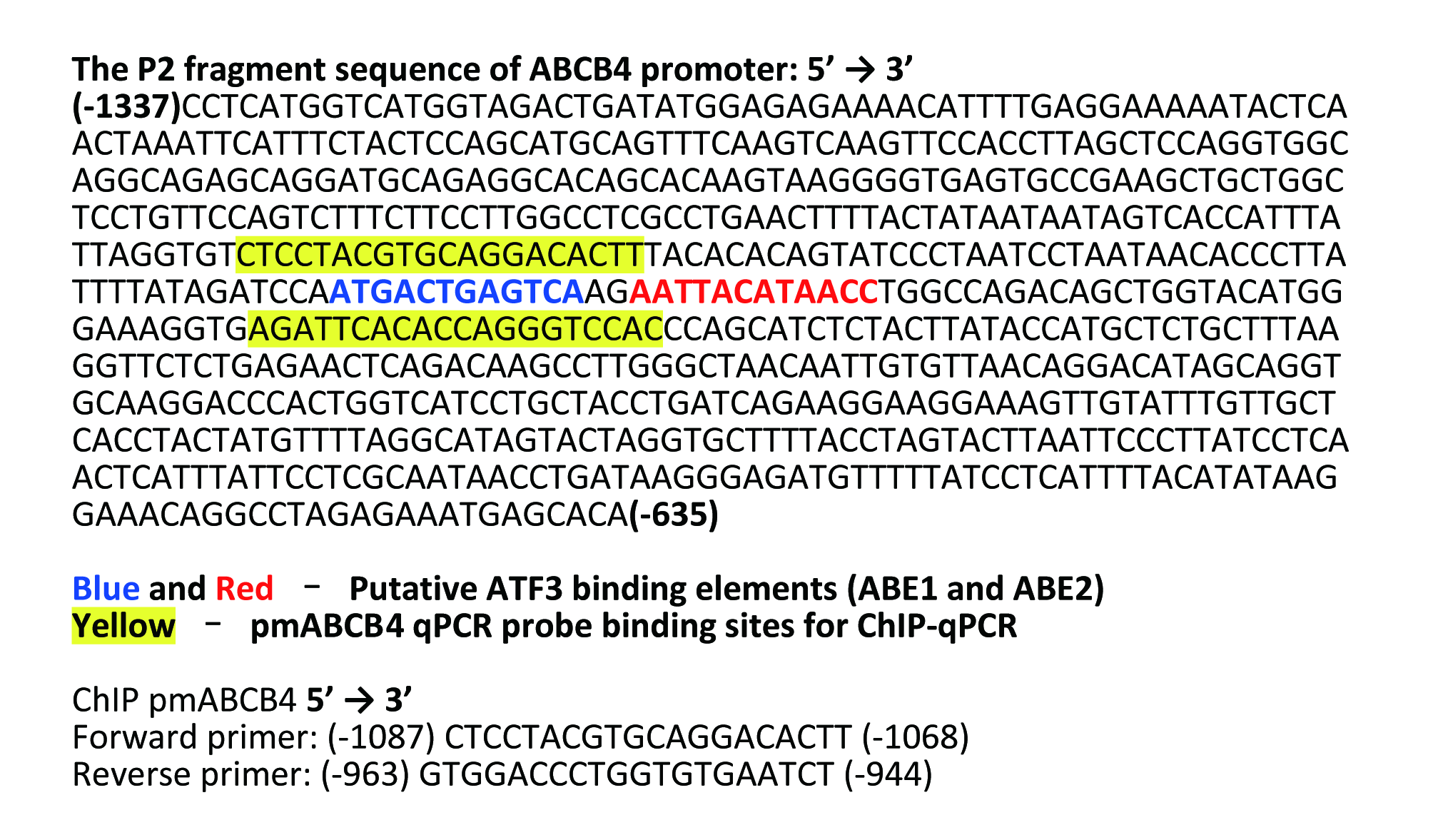


**Fig. S6 P2 fragment of the ABCB4 promoter contains two putative ABEs.** The P2 fragment sequence is as shown. Two putative ABEs are in blue and red. Yellow highlights the sequences recognized by the pmABCB4 qPCR probe used in the ChIP experiments. Specific primer sequences are shown in the figure.


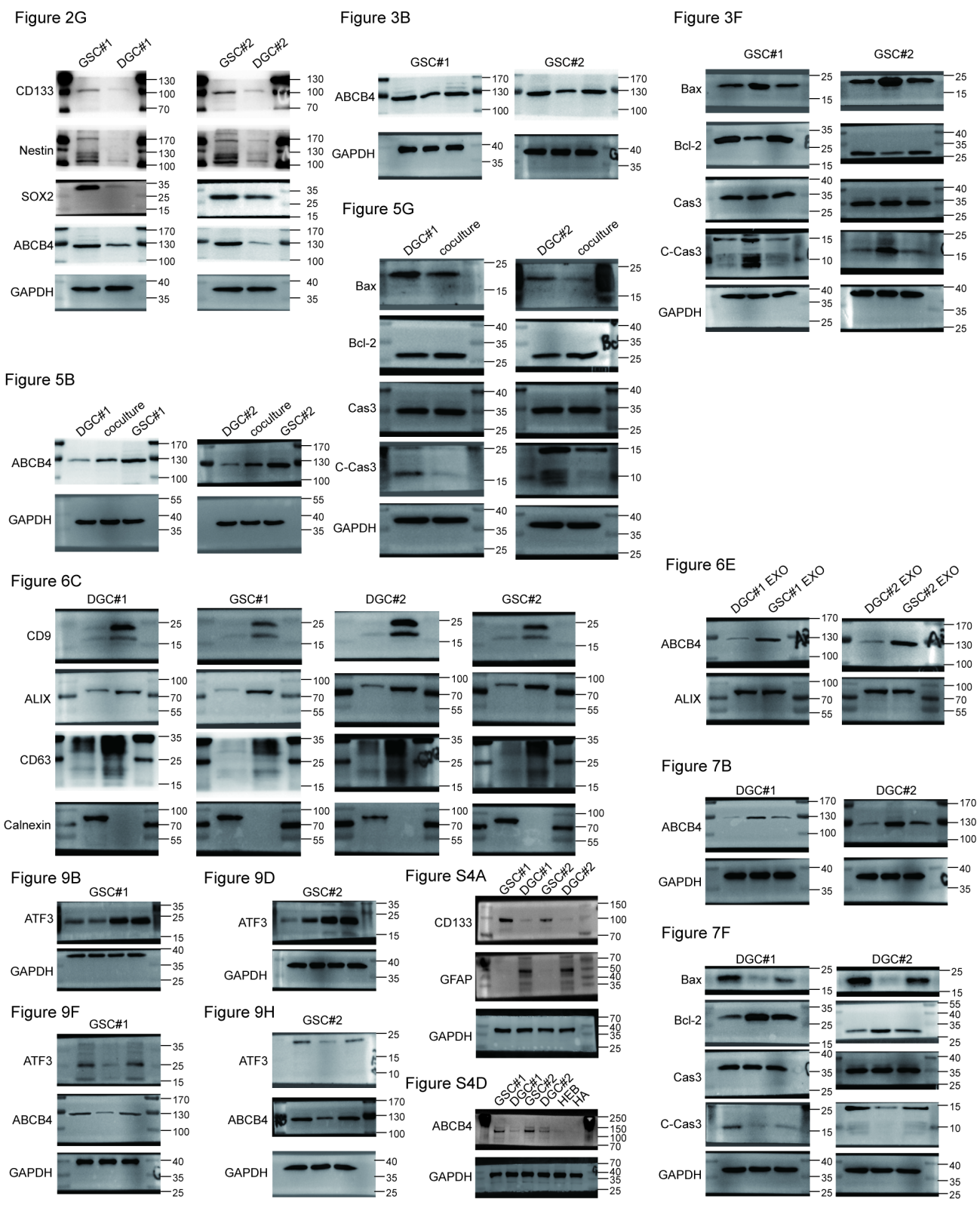


**Fig. S7 Uncropped scans of the immunoblots (Fig. 2, 3, 5, 6, 7, 9, and S4).**

| Supplemental Table 1 qRT-PCR primer sequences | |
| --- | --- |
| Genes | Primer sequences |
| GAPDH | F: 5'-GGCACCGTCAAGGCTGAGAAC-3' |
| GAPDH | R: 5'-TGCTGATGATCTTGAGGCTGTTGTC-3' |
| ABCB4 | F: 5'-TCAAATCCTCCTGTTGGATGAA-3' |
| ABCB4 | R: 5'-TTAAGTCTGCATTCTGGATGGT-3' |
| CD133 | F: 5'-CTACCAAGGACAAGGCGTTCACAG-3' |
| CD133 | R: 5'-GATGTTGGGTCTCAGTCGGTCAAG-3' |
| NEST | F: 5'-TTGAAAAAGAGACTCAACAGCG-3' |
| NEST | R: 5'-AAGATTTTACTGCCTCTACGCT-3' |
| SOX2 | F: 5'-CAGCATGTCCTACTCGCAGCAG-3' |
| SOX2 | R: 5'-CTGGAGTGGGAGGAAGAGGTAACC-3' |
| ATF3 | F: 5'-TAGGCTGGAAGAGCCAAAGA-3' |
| ATF3 | R: 5'-TTCTCACAGCTGCAAACACC-3' |
| F: forward primer, R: reverse primer | |

| **Supplemental Table 2** List of primary antibodies | | |
| --- | --- | --- |
| Antibody | Company | Cat No. |
| ABCB4 | Thermo Fisher Scientific | PA5-78692 |
| Ki67 | Abcam | ab15580 |
| cleaved caspase 3 | GeneTex | GTX86952 |
| CD133 | Cell Signaling Technology | #64326 |
| Nestin | Cell Signaling Technology | #33475 |
| SOX2 | Abcam | ab97959 |
| Bax | GeneTex | GTX109683 |
| Bcl-2 | Cell Signaling Technology | #15071 |
| caspase 3 | GeneTex | GTX13585 |
| GAPDH | Cell Signaling Technology | #3683 |
| CD9 | Cell Signaling Technology | #13174 |
| ALIX | Abcam | ab225555 |
| CD63 | Abcam | ab271286 |
| Calnexin | Abcam | ab92573 |
| ATF3 | Cell Signaling Technology | #33593 |
| GFAP | Cell Signaling Technology | #12389 |
|  | | |
